# Myeloid Tribbles 1 induces early atherosclerosis via enhanced foam cell expansion

**DOI:** 10.1126/sciadv.aax9183

**Published:** 2019-10-30

**Authors:** Jessica M. Johnston, Adrienn Angyal, Robert C. Bauer, Stephen Hamby, S. Kim Suvarna, Kajus Baidžajevas, Zoltan Hegedus, T. Neil Dear, Martin Turner, The Cardiogenics Consortium, Heather L. Wilson, Alison H. Goodall, Daniel J. Rader, Carol C. Shoulders, Sheila E. Francis, Endre Kiss-Toth

**Affiliations:** 1 Department of Infection, Immunity and Cardiovascular Disease, University of Sheffield, Beech Hill Road, Sheffield S10 2RX, UK.; 2 Division of Cardiology, Department of Medicine, Columbia University Medical Center, New York, NY 10032, USA.; 3 Perelman School of Medicine at the University of Pennsylvania and Children’s Hospital of Philadelphia, Philadelphia, PA 19104-5158, USA.; 4 Department of Cardiovascular Sciences, University of Leicester and NIHR Cardiovascular Biomedical Research Unit, Glenfield Hospital, Leicester, UK.; 5 Institute of Biophysics, Biological Research Centre, Hungarian Academy of Sciences, Temesvari korut 62, Szeged H-6726, Hungary.; 6 Departments of Biochemistry and Medical Chemistry, University of Pecs, Medical School, Szigeti ut 12, Pecs H-7624, Hungary.; 7 Division of Biomedical Services, University of Leicester, Leicester, UK.; 8 Laboratory of Lymphocyte Signalling and Development, The Babraham Institute, Babraham Research Campus, Cambridge CB22 3AT, UK.; 9 Centre for Endocrinology, William Harvey Research Institute, Queen Mary University of London and the Barts and the London School of Medicine and Dentistry, London EC1M 6BQ, UK.

## Abstract

Macrophages drive atherosclerotic plaque progression and rupture; hence, attenuating their atherosclerosis-inducing properties holds promise for reducing coronary heart disease (CHD). Recent studies in mouse models have demonstrated that Tribbles 1 (Trib1) regulates macrophage phenotype and shows that *Trib1* deficiency increases plasma cholesterol and triglyceride levels, suggesting that reduced *TRIB1* expression mediates the strong genetic association between the *TRIB1* locus and increased CHD risk in man. However, we report here that myeloid-specific *Trib1* (m*Trib1*) deficiency reduces early atheroma formation and that m*Trib1* transgene expression increases atherogenesis. Mechanistically, m*Trib1* increased macrophage lipid accumulation and the expression of a critical receptor (OLR1), promoting oxidized low-density lipoprotein uptake and the formation of lipid-laden foam cells. As *TRIB1* and *OLR1* RNA levels were also strongly correlated in human macrophages, we suggest that a conserved, TRIB1-mediated mechanism drives foam cell formation in atherosclerotic plaque and that inhibiting mTRIB1 could be used therapeutically to reduce CHD.

## Teaser

Trib1 controls atherosclerotic plaque macrophage function by up-regulating OLR1, promoting foam cell formation and atherosclerosis.

## INTRODUCTION

Atherosclerosis, a progressive disease of arterial blood vessels and the main underlying cause of stroke, myocardial infarction, and cardiac death (*1*), is initiated by the conversion of plaque macrophages to cholesterol-laden foam cells (*2*) in the arterial intima (*3*). In the early-stage atherosclerotic plaque, this transformation is induced by the uptake of both low density lipoprotein-cholesterol (LDL-C) and oxidized LDL (oxLDL) (*2*, *4*), which may serve a beneficial purpose (*3*); but unrestrained, the crucial function of plaque macrophages in resolving local inflammation is compromised, and the development of unstable, advanced lesions ensues (*3*). It has been shown that foamy macrophages are not only less effective in clearing apoptotic cells (*5*), they are also more prone to apoptosis (*6*), thus increasing secondary necrosis and the release of cellular components and lipids that ultimately form the necrotic core of advanced plaques. Hence, there have been investigations into the identities of macrophage-specific proteins that induce lipid accumulation. Thus, myeloid–lipoprotein lipase (LPL), for example, has been shown to enhance the retention of LDL-C and triglyceride-rich remnant particles within the artery wall (*7*) and induce foam cell formation (*8*), while the scavenger receptor, oxidized low-density lipoprotein receptor 1 (OLR1) has been found to internalize oxLDL (*9*), promoting not only lipid accumulation and growth but also the survival of macrophage foam cells (*10*). Conversely, myeloid-*ApoE* expression has been shown to promote high-density lipoprotein (HDL)–mediated cholesterol efflux (*11*) and macrophage switching from a proinflammatory (M1) to an alternatively (M2) activated phenotype (*12*). However, substantial advances in the development of cardiovascular disease (CVD) therapeutics await the identification of an apical regulator(s) that acts in a coordinated manner on the multiple downstream processes governing lipid accumulation, as well as atherogenicity of plaque-resident macrophages.

Tribbles 1 (Trib1) has been detected in murine plaque-resident macrophages (*13*), and variants at the *TRIB1* locus have been associated with increased risk of hyperlipidemia and atherosclerotic disease in multiple populations (*14*, *15*). However, no study had examined the macrophage-specific cellular processes dependent on myeloid-specific *Trib1* (m*Trib1*) expression and how these tally with the assumed atheroprotective properties of this pseudokinase. At the whole-body level, one study has shown that *Trib1*-deficient mice have markedly reduced numbers of M2-like (F4/80^+^ MR^+^) macrophages in multiple organs, including adipose tissue (*16*). Hence, these studies strongly implicated that loss of macrophage-*Trib1* expression within the arterial wall would lead to excessive atherosclerotic plaque inflammation and/or impair inflammation resolution and promote atheroma formation. Moreover, in hepatocytes, *Trib1* suppresses very-low-density lipoprotein production and de novo lipogenesis (*15*), indicating that the association between variants at the *TRIB1* locus and atherosclerotic disease (*14*, *15*) relates to loss of TRIB1 activity.

In the current study, we found that contrary to expectations, myeloid-specific knockout (KO) of *Trib1* is atheroprotective, while m*Trib1* expression is detrimental. In brief, *Trib1* increased OLR1 RNA and protein expression, oxLDL uptake, foamy macrophage formation, and atherosclerotic burden in two distinct mouse models of human disease. The expression of these two genes, as well as those of *LPL* and *SCARB1* (which mediates selective HDL-cholesterol uptake (*17*)), is also tightly linked in human macrophages. Collectively, our studies reveal an unexpected beneficial effect for selectively silencing *Trib1* in arterial plaque macrophages.

## RESULTS

### Myeloid Trib1 increases atherosclerosis burden

**Fig. 1:**
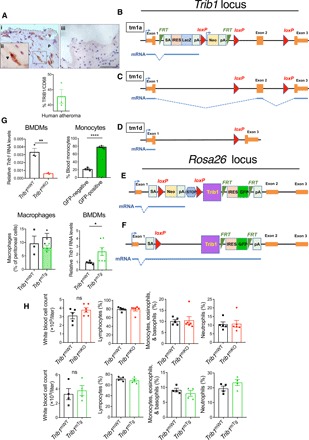
Generation and characterization of myeloid-specific *Trib1* mouse strains. (**A**) Representative immunohistochemistry image of human atherosclerotic plaque (P). Red, TRIB1; brown, CD68^+^. (ii) Magnification (×40) of boxed area. Arrowhead highlights a double-positive cell. Quantification (mean ± SD) of three patient samples. (iii) Isotype control (scale bar, 50 μm). (**B**) Targeting construct used to produce the null, conditional-ready/floxed (tm1c) and conditional-null (tm1d) *Trib1* alleles. Predicted transcripts below. FRT, flippase recognition target; SA, splice acceptor; pA, polyadenylation motif; IRES, internal ribosome entry site; LacZ, β-galactosidase; Neo, neomycin resistance gene. (**C**) *Trib1*^mWT^ allele following removal of the “gene-trap” cassette. (**D**) Conditional-null *Trib1* allele was produced by crossing tm1c and Cre-expressing mice. (**E**) Construct used to produce *Trib1*^mWT^ and *Trib1*^mTransgenic (Tg)^ mice. (**F**) Cre-mediated excision of the STOP cassette produces a bicistronic *Trib1*-*eGFP* transcript. Bent arrow, indicates transcription from endogenous *Rosa26* promoter. (**G**) *Trib1* RNA (relative to *Actb*) in BMDMs from homozygous tm1c (i.e., *Trib1*^mWT^) and *Trib1*^mKO^ mice (*n* = 3 per group). eGFP expression in monocytes of three *Trib1*^mTg^ mice and peritoneal macrophages from specified mice (*n* = 3 per group). *Trib1* RNA levels (relative to *Trib1*^mWT^) in BMDMs of *Trib1*^mTg^ (*n* = 5 to 7 per group). (**H**) Blood cell counts of mixed-gender *Trib1*^mKO^ (top) and *Trib1*^mTg^ (bottom) and their respective WT littermates (*n* = 5 to 6 per group). Data are means ± SEM. Significances were determined by Student’s *t* test, *P < 0.05, **P < 0.01, and ****P < 0.0001. ns, non-significant.

Immunostaining of human coronary atheromas from patients undergoing endarterectomies detected Trib1 in the arterial wall, including in 42.79 (± 2.31)% of CD68^+^ macrophages (Fig. 1A). We therefore examined the impact of macrophage Trib1 expression on atherogenesis by creating mice expressing low, wild-type (WT), and elevated levels of myeloid *Trib1* as outlined in Fig. 1 (B to F). Although previous studies have demonstrated that global *Trib*1 KO significantly increases perinatal lethality (*16*), *Trib1*-floxed mice and myeloid-specific Trib1 KO (*Trib1*^mKO^) mice were fully viable and bred normally (fig. S1, A to D). m*Trib1* transgenic (overexpressing, *Trib1*^mTg^) mice were also fully viable and bred normally. m*Trib1* RNA levels were substantially lower in *Trib1*^mKO^ than in floxed WT littermates, as judged by reverse transcription–quantitative polymerase chain reaction (RT-qPCR) assays performed on bone marrow–derived macrophages (BMDMs) prepared from these animals (Fig. 1G). As judged by enhanced green fluorescent protein (eGFP) expression, the bicistronic *Trib1* transgene was expressed in 78.43 ± 2.33% and 65.58 ± 0.92% of blood monocytes and peritoneal macrophages, respectively (Fig. 1G), and overall, the transgene increased BMDM *Trib1* RNA levels by 2.49 ± 0.43 (SEM) fold (Fig. 1G, bottom right). Consistent with previous findings (*18*), the transgene was also expressed in neutrophils, which form a minor component of the immune cell population within very early-stage atherosclerotic lesions (*19*). Thus, we detected eGFP in 53.88 ± 2.41% and 34.93 ± 2.96% of *Trib1*^mTg^ blood and bone marrow CD11b^+^/Ly6C^−^/Ly6G^+^ cells, respectively, compared to 25.95 ± 3.16% and 12.42 ± 2.01% in their CD11b^+^/Ly6C^+^/Ly6G^−^ monocyte counterparts (fig. S1, E to I). However, in marked contrast to the reported full-body *Trib1* KO mouse (*16*), *Trib1*^mKO^ mice were not afflicted by reduced numbers of total, or individual, white blood cells (Fig. 1H) or by reduced macrophage numbers in their adipose tissue (F4/80^+^, fig. S2A), liver (F4/80^+^, fig. S2B), or spleen (F4/80^+^ and CD206^+^, fig. S2C). Similar to *Trib1*^mKO^ mice, *Trib1*^mTg^ mice displayed no gross abnormalities and had WT numbers of white blood cells (Fig. 1H). In addition, the sizes of their adipocytes (fig. S2A), liver (fig. S2B), and splenic (fig. S2C) macrophage populations were unaltered.

**Fig. 2:**
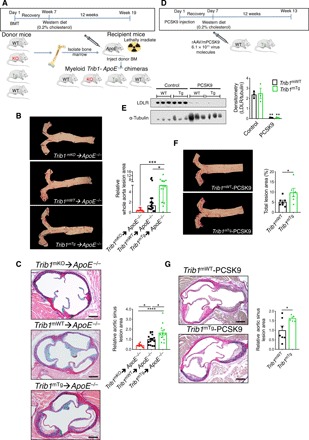
Myeloid-specific *Trib1* expression increases atherosclerosis burden in two murine models of human atherosclerosis. (**A**) Schematic of the bone marrow transplant experiment. Bone marrow cells from myeloid-specific *Trib1* KO and transgenic (Tg) mice and their respective WT controls were transplanted into *ApoE*^−/−^ recipients. (**B**) Representative en face Oil Red O staining of thoracic aortas (week 19) from specified chimeras. Lesion areas were calculated as percentages of the total surface area of the whole aorta and normalized (median, ±95% confidence interval) to *Trib1*^mWT^; *n* = 10 to 18 per group. (**C**) Representative images of Elastic van Gieson–stained aortic sinus lesions. Quantification relative to WT (*n* = 10 to 16 mice per group). (**D**) Second model of human atherosclerosis. rAAV/mPCSK9, recombinant adenovirus–produced murine proprotein convertase subtilisin/kexin 9. (**E**) LDLR protein in liver samples from specified mice was quantified by Western blotting (*n* = 3 per group). (**F**) Representative en face Oil Red O staining of thoracic aortas from specified mice. Lesion areas were calculated as percentages of the total surface areas of the whole aorta (*n* = 6 to 7 per group). (**G**) Representative images of Elastic van Gieson–stained aortic sinus lesions of specified mice and quantification, relative to WT (*n* = 5 to 7 per group). Scale bars, 200 μm (C and G). Data are means ± SEM. Significance was determined by one-way (B and C) or two-way analysis of variance (ANOVA) (E) or Student’s *t* test (F and G). *P < 0.05, **P < 0.01, ***P < 0.001, and ****P < 0.0001.

To address the contribution of m*Trib1* in early atherosclerosis, we first transplanted bone marrow cells from the *Trib1*^mKO^ and *Trib1*^mTg^ mice and their respective controls (i.e., non-CRE, floxed KO, and Tg alleles) into 12- to 13-week-old lethally irradiated male *ApoE*^−/−^ mice (Fig. 2A). Thus, all recipient mice received *ApoE^+/+^*–bone marrow cells to mitigate the previously described effects of total ablation of this apolipoprotein on both classical/proinflammatory (M1) and alternative/anti-inflammatory (M2) polarization (*12*) and to provide them with a physiologically important source of APOE to aid normalization of plasma cholesterol levels and of the lipoprotein profile in this otherwise extreme hyperlipidemic mouse model of human atherosclerosis (*12*, *20*). In short, this experiment allowed modeling of early atherogenesis in a setting more akin to the human disease. Following a 7-week recovery period, the chimeric mice were fed a Western diet containing 0.2% cholesterol for 12 weeks. At sacrifice, and consistent with expectations of the study design, the chimeric mice had relatively low plasma cholesterol levels for a mouse model of human atherosclerosis (fig. S3A).

Unexpectedly, we found less atherosclerosis in the thoracic aorta of *Trib1*^mKO^➔*ApoE*^−/−^ chimeras than in the control WT mice (Fig. 2B and fig. S4A). Conversely, there was a significantly higher atheroma burden in the *Trib1*^mTg^➔*ApoE*^−/−^ mice (Fig. 2B and fig. S4A). Similarly, the lesions in the aortic sinus were, on average, smaller in the *Trib1*^mKO^➔*ApoE*^−/−^ mice and larger in the *Trib1*^mTg^➔*ApoE*^−/−^ mice (Fig. 2C and fig. S4B). However, the collagen contents of the *Trib1*^mKO^➔*ApoE*^−/−^ and *Trib1*^mTg^➔*ApoE*^−/−^ in these “early-stage” plaques and their clinical pathology were comparable to those of the chimeric *Trib1*^mWT^➔*ApoE*^−/−^ mice (fig. S4C). In short, we found that m*Trib1* expression increased the atherosclerotic burden of *ApoE*^−/−^ mice, despite having little impact on plasma LDL-cholesterol levels (fig. S3A).

To confirm that m*Trib1* accelerates the development of atherosclerosis (Fig. 2, B and C), we created an LDL receptor (*Ldlr*) knockdown model of human atherosclerosis (*21*) to induce hyperlipidemia and atherosclerosis in otherwise WT mice (*21*). Specifically, mice were injected with an adeno-associated virus (rAAV8) encoding for proprotein convertase subtilisin/kexin 9 (Fig. 2D), which previous studies have shown lowers both hepatic and extrahepatic surface cell expression of the *Ldlr* (*22*). Following feeding a Western diet for 12 weeks, this intervention produced comparable, highly significant reductions in LDLR protein levels in the *Trib1*^mTg^ and *Trib1*^mWT^ mice (Fig. 2E) and a similar degree of hyperlipidemia (fig. S3B). However, despite this and consistent with m*Trib1* expression increasing atheroma formation in *ApoE^−/−^* mice, *Trib1*^mTg^ injected with rAAV8-Pcsk9 developed a significantly higher atherosclerotic burden in their aorta and aortic sinus than their similarly injected *Trib1*^mWT^ mice (Fig. 2, F and G).

### Myeloid Trib1 increases macrophage/foam cell size in the atherosclerotic plaque

**Fig. 3:**
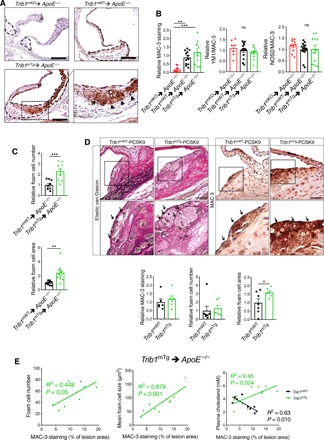
Myeloid-Trib1 induces foam cell expansion. (**A**) MAC-3 staining (brown) of representative cross sections of the aortic sinus from specified mice (scale bars, 100 μm). Dashed lines indicate lesion boundaries. Arrows highlight foam cells in the boxed region (40-fold magnification). (**B**) Staining of aortic sinus lesions from specified chimeric mice with specified antibodies: MAC-3 (*n* = 9 to 12 per group) and double-positive YM1/MAC-3 (*n* = 7 to 14 per group) and NOS2/MAC-3 (*n* = 10 to 16 per group) cells. (**C**) Quantification of relative foam cell numbers (top) and size (bottom) in aortic sinus lesions of specified chimeras. *n* = 10 to 16 per group. (**D**) Representative images (scale bars, 30 μm) of Elastic van Gieson– and MAC-3–stained aortic sinus lesions from specified mice injected with rAAV/mPCSK9 (*n* = 9 to 11 per group). Arrows in magnified (×40) images of boxed area highlight foam cells. Quantification of MAC-3 staining and foam cell numbers and sizes (*n* = 6 to 7 per group). (**E**) Correlation between MAC-3 staining (*x* axis) in aortic sinus lesions of specified chimeric mice and foam cell numbers (left), foam cell size (middle), and plasma cholesterol levels (right). MAC-3 staining expressed as percentage (%) of total aortic sinus lesion area. *R*^2^, Pearson correlation coefficient. In (B) to (D), data (means ± SEM) are expressed relative to WT. Statistical differences were determined by one-way ANOVA (B) or Student’s *t* test (C and D). *P < 0.05, **P < 0.01, and ***P < 0.001.

Next, we investigated the macrophage content and phenotype in the atherosclerotic plaque in each mouse model. This revealed that the aortic sinus lesions of *Trib1*^mKO^➔*ApoE*^−/−^ mice contained a much smaller MAC-3^+^ immunoreactive area than the chimeric *Trib1*^mWT^➔*ApoE*^−/−^ mice, while on average, the *Trib1*^mTg^➔*ApoE*^−/−^ atheromas contained a marginally larger stained area (Fig. 3, A and B). However, there was no preferential loss of YM1^+^ macrophages in the *Trib1*^mKO^➔*ApoE*^−/−^ lesions (Fig. 3B), consistent with the finding that M2 polarization of *Trib1*-deficient BMDMs isolated from whole-body *Trib1*^mKO^ mice are compromised to a similar extent as M1 polarization (*23*). In addition, we could not attribute the proatherogenic activity of myeloid *Trib1* expression to a preferential increase in the proinflammatory macrophage (NOS2^+^) content of *Trib1*^mTg^➔*ApoE*^−/−^ plaque (Fig. 3B, right). Rather, the increased atherosclerosis in the *Trib1*^mTg^➔*ApoE*^−/−^ chimeras was attributable to a doubling of foam cell numbers (cells with characteristic foamy appearance and MAC-3^+^ (fig. S4D), and on average, these cells were also larger (Fig. 3, A and C, and fig. S4D). While the Trib1^mWT^-Pcsk9 atheromas had a similar-sized macrophage population (Fig. 3D), the size of the plaque-resident foam cells in the transgenic animals was larger (Fig. 3D).

In the *Trib1*^mTg^➔*ApoE*^−/−^ chimeras, there was a stronger correlation between the mean foam cell size and the percentage of aortic sinus stained by MAC-3 than between MAC-3^+^ staining and foam cell numbers (Fig. 3E), and we could not ascribe the observed increase in plaque-foam cell numbers on the effects of m*Trib1* expression on blood cholesterol levels (Fig. 3E), HDL-C levels, or the nonsignificant rise in LDL-C (fig. S3). While the *Trib1*^mWT^➔*ApoE*^−/−^ chimeras with the highest plasma cholesterol, HDL-C, and LDL-C concentrations had the lowest amount of MAC-3^+^ staining in their aortic sinus lesions, the inverse was true for the *Trib1*^mTg^➔*ApoE*^−/−^ chimeras (Fig. 3E, right, and fig. S3). Thus, collectively, these data indicate that increased macrophage lipid uptake/storage was the prominent driving force for the observed foam cell expansion besetting the early stage of the atherosclerotic process in these and the *Trib1*^mTg^*-Pcsk9* mice.

### Myeloid TRIB1 expression induces OLR1 expression in both mouse and man

**Fig. 4:**
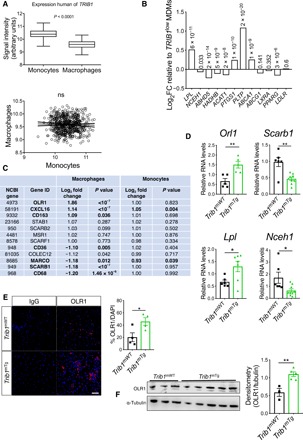
Myeloid TRIB1 induces reciprocal changes in oxLDL and HDL receptor expression in human and mouse macrophages. (**A**) *TRIB1* RNA levels in monocytes and MDMs of CTS participants (*24*). Correlation (*R*^2^ < 0.001, *P* = 0.47) was performed on 596 paired monocyte and MDM samples. (**B**) MDM (*n* = 596) and monocytes (*n* = 758) were ranked according to *TRIB1* RNA contents and gene expression values in the top and bottom quartiles compared. Log_2_ fold changes (FC) of specified RNAs in *TRIB1*^High^ (*n* = 149) versus *TRIB1*^Low^ (*n* = 149) MDM, with associated *P* values. (**C**) FC and *P* values for differential expression of RNAs encoding representative scavenger receptors, including CD36, which mediates (ox)-phospholipid and long-chain fatty acid uptake (*39*), the acetylated-LDL scavenging receptor (*40*), and macrophage scavenger receptor (*40*). Comparisons are between *TRIB1*^High^ versus *TRIB1*^Low^ MDMs and between *TRIB1*^High^ (*n* = 191) versus *TRIB1*^Low^ (*n* = 191) monocytes. (**D**) RT-qPCR quantification of RNA (mean ± SEM) in BMDM prepared from specified mice (*n* = 5 to 9 per group). (**E**) Immunocytochemistry of nonpolarized *Trib1*^mWT^ and *Trib1*^mTg^ BMDMs. OLR1 (red), nuclei counterstained with 4′,6-diamidino-2-phenylindole (DAPI) (blue). Scale bar, 50 μm. Quantifications performed on BMDMs prepared from four to five mice per group. (**F**) Western blot analysis of OLR1 in *Trib1*^mWT^ and *Trib1*^mTg^ BMDMs (*n* = 3 to 5 per group). In (D) to (F), significance was determined by Student’s *t* test; *P < 0.05, and **P < 0.01.

To identify potential cellular mechanisms by which m*Trib1* enhances foam cell expansion, we analyzed the gene expression characteristics of human *TRIB1*^High^ monocytes and *TRIB1*^High^ monocyte-derived macrophages (MDMs) using the microarray RNA data produced in the Cardiogenics Transcriptomic Study (CTS) (*24*). In this dataset, involving samples from 758 individuals, *TRIB1* RNA levels were, on average, higher in monocytes than in MDMs (Fig. 4A, top) but, as is evident from the analyses of RNA levels in 596 paired samples, there was no correlation between *TRIB1* RNA levels in these two cell types (Fig. 4A, bottom). Moreover, genes differentially expressed in *TRIB1*^High^ versus *TRIB1*^Low^ monocytes were enriched for different sets of “DAVID” Gene Ontology cluster terms (tables S1 and S2) than those characterizing the more lipid-based transcriptome of *TRIB1*^High^ MDM (Fig. 4, B and C, and table S3).

The Cardiogenics Transcriptomic data strongly suggested that the m*TRIB1*-induced foam cell phenotype stemmed from increased oxLDL uptake rather than changes in LDLR and scavenger receptor class B type 1 [which mediates selective HDL-cholesterol uptake and efferocytosis (*17*)] expression (Fig. 4C) or reductions in ABCG1- and ABCA1-mediated cholesterol efflux (Fig. 4B). Notably, *OLR1* was the fourth most differentially expressed gene in the *TRIB1*^High^ human MDMs and the most highly altered scavenger receptor in these cells (Fig. 4C). We therefore examined the effect of m*Trib1* transgene expression on the expression of this oxLDL receptor (*9*). This revealed that *Trib1*^mTg^ BMDMs contained more *Olr1* RNA but fewer *Scarb1* transcripts (Fig. 4D) than their *Trib1*^mWT^ counterparts, indicating that the increased numbers of *OLR1* and reduced numbers of *SCARB1* transcripts in human *TRIB1*^High^ MDMs are causally related to the increased number of *TRIB1* transcripts in these cells. The reciprocal relationship between *OLR1* and *SCARB1* RNA levels in *TRIB1*^High^ MDMs was also recapitulated in interferon-γ (IFN-γ)/lipopolysaccharide (LPS), interleukin-4 (IL-4)–polarized (fig. S5A) and fatty acid–polarized MDM samples but not in HDL-polarized MDMs (fig. S5B and table S4). Rather, HDL-polarized MDMs contained *OLR1* and *SCARB1* RNA levels indistinguishable from those of nonpolarized MDMs (fig S5, A and B, and table S4), indicating that *Trib1* induces Olr1 expression and foam cell formation via a non-HDL uptake–mediated mechanism. Last, to substantiate the evidence for causal TRIB1 involvement in OLR1 expression, we stained BMDMs for OLR1 protein. OLR1 was detected in twice as many *Trib1*^mTg^ cells than their WT counterparts (Fig. 4E), consistent with the Western blotting analysis of whole BMDM cell lysates (Fig. 4F).

### m*Trib1-*induced OLR1 expression in plaque macrophages increases atherosclerotic burden

**Fig. 5:**
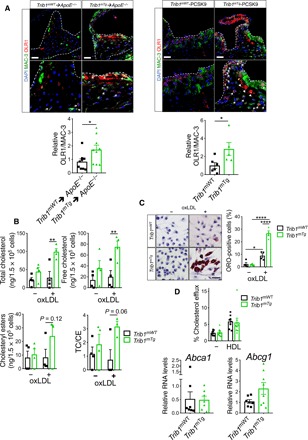
Myeloid-Trib1 increases ORL1 expression, cholesterol uptake, and neutral lipid accumulation. (**A**) Representative images (scale bars, 25 μm) of aortic sinus lesions from specified mice and enlarged images. Dashed lines indicate boundaries of lesions. MAC-3 (green), OLR1 (red), and nuclei counterstained with DAPI (blue). Arrows indicate OLR1-positive macrophages. Arrowheads indicate assumed acellular OLR1. Quantification: *Trib1*^mWT^➔*ApoE^−/−^* and *Trib1*^mTg^➔*ApoE^−/−^* chimeras, nine per group; m*Trib1*-PCSK9, five to six per group (mean ± SEM). (**B**) Intracellular total cholesterol, unesterified cholesterol, and cholesteryl ester contents of *Trib1*^mTg^ and *Trib1*^mWT^ bone marrow cells differentiated into macrophages and incubated with oxLDL (25 μg/ml) for 24 hours (*n* = 4 per group). (**C**) Representative images of BMDMs stained with Oil Red O (scale bar, 50 μm). Quantification was performed on three fields of view per sample. (**D**) Quantification of cholesterol efflux from cholesterol-loaded BMDMs to human HDL (*n* = 8 to 9 per group). RT-qPCR quantification of *Abca1* and *Abcg1* RNA in nonpolarized BMDMs prepared from specified mice (*n* = 7 to 8 per group). Data are means ± SEM. Significance determined by Student’s *t* test (A and D, bottom panels) or two-way ANOVA with Sidak’s multiple comparisons posttest (B to D) *P < 0.05, **P < 0.01, and ****P < 0.001.

To corroborate the evidence for causal mOLR1 involvement in plaque-resident macrophage foam cells expansion, we quantified OLR1 expression in the aortic sinus lesions of our mouse models using an OLR1 antibody that recognizes the cell surface–expressed form of this oxLDL receptor, as well as the proteolytically cleaved (soluble) extracellular form (*9*). The antibody detected OLR1 in MAC-3^+^ cells and in acellular areas of the mouse aortic sinus lesions, including in regions adjacent to plaque macrophages (Fig. 5A). Moreover, as expected from the known expression and regulation of this scavenger receptor in endothelial cells (*9*), significant amounts of OLR1 was also detected in the nonmacrophage (i.e., MAC-3^−^) cell population at the plaque surface of the more hyperlipidemic model of human atherosclerosis (Fig. 5A and fig. S3). Last, confirming the causal involvement of mTRIB1 in mOLR1 expression (Fig. 4, D to F), Fig. 5A shows that the anti-OLR1 antibody detected OLR1 in more of the plaque macrophages of *Trib1*^mTg^➔*ApoE*^−/−^ mice than in those of the *Trib1*^mWT^➔*ApoE*^−/−^ control animals (33.89 ± 6.56% versus 16.18 ± 4.05%); a result which was replicated in the *Trib1*^mTg^-*Pcsk9* and *Trib1*^mWT^-*Pcsk9* mice (29.35 ± 7.42% versus 10.38 ± 3.36%) (Fig. 5A).

To mechanistically validate the contribution of m*Trib1*-induced Olr1 expression to foam cell expansion, we incubated nonpolarized BMDMs with oxLDL for 24 hours. This led to marked rises in the intracellular levels of both total cholesterol (2.71 ± 0.24–fold, *P* = 0.0091) and unesterified cholesterol (5.81 ± 0.83–fold, *P* = 0.0049) in the *Trib1*^mTg^ BMDMs but not in their WT counterparts (Fig. 5B). In addition, as judged by Oil Red O staining, oxLDL transformed nearly three times as many *Trib1*^mTg^ BMDMs into foam cells than *Trib1*^mWT^ cells (*P* < 0.0001, Fig. 5C), as evidenced by the very visible increase in neutral lipid accumulation in these cells upon exposure to oxLDL. In contrast to the profound effects of oxLDL on cholesterol accumulation in unpolarized *Trib1*^mTg^ BMDMs, we observed no impairment of HDL-mediated cholesterol efflux (Fig. 5D), consistent with the observation that neither *Abca1* nor *Abcg1* RNA levels are reduced in these BMDMs (Fig. 5D) and that the *Trib1*^mTg^➔*ApoE*^−/−^ chimeras have higher, rather than lower, HDL-C levels than their WT peers (fig. S3). Thus, collectively, our results suggest that *Trib1*-induced foam cell expansion in early-stage atherosclerotic plaque arises from increased cholesterol/neutral lipid uptake and retention rather than reduced HDL-mediated cholesterol efflux.

## DISCUSSION

**Fig. 6:**
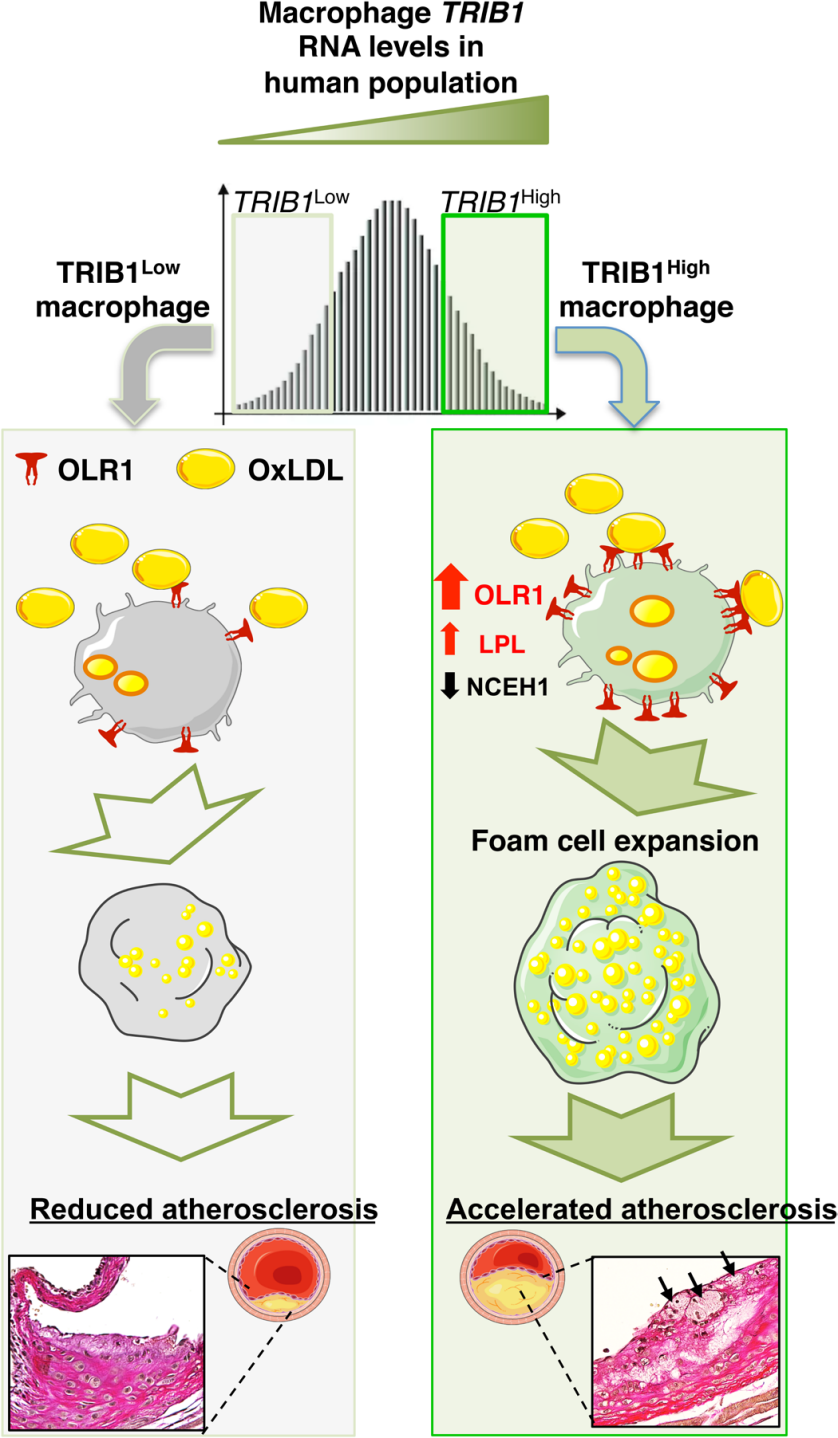
Model summarizing the proposed effects of differences in mTRIB1 expression on foam cell expansion in early-stage atherosclerosis. Factors up-regulating m*TRIB1* expression in human MDMs and plaque macrophages simultaneously increase cholesterol and neutral lipid uptake, with no compensatory rise in cholesterol efflux. Schematic recognizes that increased OLR1 expression increases the probability of this scavenger receptor assembling as a hexamer made up of three homodimers on the macrophage cell surface and that this configuration leads to a marked increase in its affinity for oxLDL (*9*) and hence OLR1-mediated uptake of oxLDL lipids. In the setting of no compensatory rise in HDL-mediated cholesterol efflux, accelerated foam cell expansion and increased atheroma burden ensue, highlighting the therapeutic potential of inhibiting macrophage Trib1 expression to block the gene expression changes that both promote macrophage cholesterol uptake and cholesteryl ester formation accumulation and prevent the increased hydrolysis of the accumulated cholesteryl ester and thus the up-regulation of the reverse cholesterol transport pathway to mediate the removal of cholesterol from the arterial wall.

Despite the success in establishing that hepatic *Trib1* expression affects the regulation of multiple cellular processes modulating blood cholesterol and triglyceride levels (*15*), the influence of global-KO of *Trib1* on shaping the phenotype of macrophages (*16*), and the finding that variants at the *TRIB1* locus are associated with and increased coronary heart disease (CHD) risk (*14*), the contribution of Trib1 on atherogenesis remains to be addressed. Here, we demonstrate that there is a wide distribution of *TRIB1* RNA levels in human MDMs and that genetically engineered changes in m*Trib1* expression in mouse models of early-stage human atherosclerosis markedly affect the size of developing plaques and the morphological and functional properties of plaque macrophages (Fig. 6). In summary, we have confirmed the proatherogenic impact of myeloid TRIB1 in two distinct in vivo models of human atherosclerosis.

In the experiments reported here, we provide evidence that m*Trib1* transgene expression may reduce vascular cell exposure to oxLDL given that it increased the size and lipid contents of plaque-resident foam cells in two independent models of early-stage atherosclerosis by increasing mOLR1 expression and oxLDL uptake. Notably, in the less hyperlipidemic of these two transgenic models, we could also discern a very strong positive correlation between plaque macrophage numbers and plasma cholesterol/LDL-C levels, implying that in early-stage atherosclerosis, m*Trib1*^High^ macrophages (in marked contrast to Trib1-deficient macrophages) are uniquely equipped to increase plaque macrophage numbers in response to lipid excess and hence foam cell and fatty streak formation.

Recent studies have established that oxLDL accumulates steadily in both early-stage (growing) and mature-stage (yellow plaque without a necrotic core) human coronary plaques but that, in more advanced vulnerable plaques (yellow plaques with a necrotic core), this lipoprotein is removed either by metabolism or replacement with other substances, including cell debris (*2*). These in vivo data dovetail well with the early in vitro work, which showed that while oxLDL promotes macrophage growth and survival in a dose-dependent manner, beyond a certain lipid concentration, cell death ensues, albeit by an unknown mechanism (*10*). Thus, a critical question to consider is whether m*TRIB1*-induced OLR1 expression serves an (athero-) protective role in human atherosclerosis, for example, by reducing the exposure of plaque-resident vascular cells (where this disease is initiated) to oxLDL. This lipoprotein is a well-described activator of endothelial cell OLR1 expression with the totality of the data indicating that this activation culminates in arterial endothelium dysfunction (*9*). That said, the endothelium is activated by numerous factors, including blood flow, hypertension, and inflammation (*25*), which over the life span of an individual may attenuate (and even erase) the potential atheroprotective effect arising from an m*TRIB1*-mediated reduction in the exposure of endothelial cells to oxLDL. By contrast, the effects of an mTRIB1-induced increase in OLR1 expression will directly bear on atherosclerotic disease progression. Notably, it has been shown that mice overexpressing *Olr1* on an *ApoE* KO background develop markedly more atherosclerosis than their nontransgenic littermates (*26*) and that *Olr1* deficiency on an *Ldlr* KO background reduces the atherogenic burden conferred by 18 weeks of a 4% cholesterol/10% cocoa butter diet (*27*). With specific reference to the macrophage component of atheromas, Schaeffer and colleagues (*28*) have shown that cytokine-induced up-regulation of OLR1 increased OxLDL internalization by >40%, suggesting that (in line with our data presented in fig. S5), in inflamed microenvironments, where proinflammatory cytokines are relatively abundant (such as in atherosclerotic lesions), increased OLR1 expression could play a substantial role in up-regulating oxLDL uptake and atheroma formation. Supporting this contention, we show here that the TRIB1-induced increase in OLR1 expression increased the uptake of oxLDL and lipid accumulation in plaque macrophages, which in vitro (*10*) and in vivo (*9*) studies have indicated, all other things being equal, will accelerate the formation of foam cells, foam cell apoptosis/necrosis, and the development of more advanced, complex lesions.

On the basis of the strong evidence of a causal link between hyperlipidemia and CHD (*29*) and the demonstration that ablating hepatic *TRIB1* expression increased plasma levels of cholesterol, LDL-C, and triglyceride (*15*), the implication was, which seemed to be entirely consistent with results from genome-wide association studies (GWAS) (*14*), that increasing *TRIB1* would be atheroprotective. Silencing *TRIB1* expression in macrophages, however, turns out to be atheroprotective, as judged by analyses of the aortas and aortic sinuses from *Trib1*^mKO^➔*ApoE*^−/−^ chimeric mice, after a 12-week Western dietary regime. This counterintuitive result, which fits well with our in vitro and in vivo analysis of the consequences of m*Trib1*^High^ expression on foam cell expansion, suggests moving forward that developing a therapy to specifically silence *Trib1* expression in macrophages would provide clinical benefit beyond that of lipid-lowering medications, although full realization of this benefit may require its adoption at an early stage of atherogenesis. More generally, our study also demonstrates that mechanistic probing of GWAS signals is not only warranted, but also critical, to identify both the totality and directionality of disease risk factors, even when, as was the case for *TRIB1*, the association between a genetic variant(s), disease risk and a major disease-risk factor (plasma lipids) appeared congruent. Hence, we acknowledge that a limitation of the current study is that we have not addressed whether changes in vascular cell *TRIB1* expression might affect early-stage atherosclerosis development and whether the GWAS-CHD signal at the *TRIB1* locus reflects that, in these cells (and hepatocytes), TRIB1 serves an atheroprotective role, in contrast to the situation in plaque-resident macrophages, where it induces foam cell expansion.

One of the most notable outcomes arising from roughly doubling *Trib1* expression in macrophages was the increase in foam cell size that developed in both of our models of human atherosclerosis. Our results indicate that this was driven by the failure of these cells to increase HDL-mediated cholesterol efflux in response to an up-regulation of cholesterol and fatty acid uptake. In addition, this up-regulation was attributable to increased OLR1 RNA and protein expression, consistent with the in vitro studies of Lazar and co-workers (*30*), which demonstrated that Rosiglitazone induction of *Olr1* expression in adipocytes increased oxLDL and palmitate uptake and cellular cholesterol levels. Likewise, when Steinbrecher and colleagues (*28*) increased macrophage *OLR1* expression via lysophosphatidylcholine stimulation, oxLDL uptake was also induced, prompting them to introduce the concept that macrophages within atheromas may be quiescent with respect to oxLDL uptake until *Olr1* expression is induced (*28*). Here, we validate this concept by demonstrating overexpressing m*Trib1* led directly to an increase in OLR1 protein in plaque-resident macrophages, alongside their increased lipid contents and size. Our data also show that by doubling m*Trib1* expression, we removed the requirement for an external stimulus to up-regulate *OLR1* expression and the consequent clinical sequelae. The phenotype observed in response to this rise in m*Trib1* expression fits well with earlier computational modeling studies that indicated that relatively modest changes in TRIB1 would have a major impact on the activity of mitogen-activated protein kinase pathways; thus, defining the concentration of these proteins is critical for shaping cell function (*31*).

Thus, from the therapeutic standpoint, our results reveal that targeting TRIB1 expression could, in addition to beneficially affecting OLR1 expression, moderate CHD pathogenesis by simultaneously altering in favorable directions the expression of a number of other disease-promoting genes affected by changes in TRIB1 expression. These would include, for example, beneficially increasing NCEH1 expression to reduce the release of proinflammatory cytokines from plaque-resident macrophages (*32*), while reducing the expression of LPL to help reduce the retention of LDL in the artery wall during early-stage atherosclerosis (*7*), as well as excessive accumulation of cholesteryl ester and triglycerides within macrophage foam cells (*8*). Whether mTRIB1 modulates atheroma regression (*33*, *34*) and late-stage atherosclerotic plaque stability by orchestrating a coordinated response to the lipid and inflammatory challenges encountered by plaque-resident macrophages/foam cells in advanced stage atherosclerosis now requires investigation.

## MATERIALS AND METHODS

### Human samples

Human tissue and blood samples were collected under protocols approved by the University of Sheffield Research Ethics Committee and Sheffield Teaching Hospitals Trust Review Board (ref. STH 16346 and SMBRER310) and in accordance with the Declaration of Helsinki. All participants gave written informed consent. Human atheromas were from the left anterior descending artery of explanted hearts from three patients with ischemic heart disease undergoing cardiac transplantation. Patients were male and had a mean age of 55.67 ± 2.51 (SD) years at the time of surgery. Lesions were classified as type V (American Heart Association). Specimens were fixed in 10% (v/v) formalin and embedded in paraffin wax. Antigen retrieval was performed with trypsin for 15 min at room temperature (MP-955-K6, A. Menarini Diagnostics, UK). Sections were incubated with mouse anti-human CD68 (M0814, Clone KP1, Dako) antibody (1:100 dilution) and rabbit anti-human TRIB1 (09-126, Millipore, UK) antibody (1:100) and the appropriate secondary antibodies, biotinylated horse anti-mouse secondary antibody and goat anti-rabbit secondary (BA-2000 and BA-1000, Vector Laboratories) antibody (both 1:200 dilution); and the detection reagents, Elite Mouse ABC horseradish peroxidase (HRP) (PK-6100, Vector Laboratories), 3, 3′-diaminobenzidine (SIGMA*FAST*, D4293, Sigma-Aldrich), and rabbit ABC-Alkaline phosphatase and a Vector Red Alkaline phosphatase substrate kit (AK-5000 and SK-5100, Vector Laboratories). Sections were counterstained with hematoxylin and mounted with DPX mountant (44581, Sigma-Aldrich).

### Mice and creation of murine models of myeloid-specific *Trib1* expression

Mice were handled in accordance with U.K. legislation (1986) Animals (Scientific Procedures) Act. Mouse experiments were approved by the University of Sheffield Project Review Committee and carried out under a U.K. Home Office Project Licence (70/7992). All mice used were congenic on a C57BL/6J background (N17) and were housed in a controlled environment with a 12-hour light/dark cycle, at 22°C in Optimice® individually ventilated cages (Animal Care Systems) and given free access to a standard chow diet (2918; Harlan Teklad) and water. A knockout mouse project (KOMP) repository embryonic stem (ES) cell clone containing loxP sites flanking exon 2 of *Trib1* (EPD0099_5_D04) was used to generate a floxed *Trib1* allele. The clone was genotyped to validate its authenticity, injected into C57BL/6J blastocysts, and transferred to pseudopregnant recipient females (Geneta). Resulting chimeras were mated with a flippase (FLP)–deleter strain maintained on a C57BL/6J background (Geneta), and floxed-*Trib1* mice were generated. *Trib1*^mKO^ mice were generated by crossing floxed mice with Lys2–cre recombinase transgenic mice (www.jax.org/strain/004781), excising all but the first 120 amino acids of TRIB1. Murine *Trib1* complementary DNA (cDNA) was introduced into the previously described pROSA26, loxP-flanked STOP and Frt-flanked internal ribosomal entry site–eGFP targeting construct (*35*) and then inserted into the ubiquitously expressed *Rosa26* locus of Bruce4 (C57/BL6J origin) mouse ES cells by homologous recombination. Correct integration was confirmed by Southern blotting. *Trib*^1mTg^ mice were generated by crossing floxed mice with the Lys2–cre recombinase transgenic mice described above. Mice were genotyped by PCR amplification of ear-clip samples. *Trib1 fl/fl* × *Lyz2Cre* and *Rosa26.Trib1* × *Lyz2Cre* were genotyped for the presence of *Lyz2Cre* and for either *Trib1 fl/fl* or *Rosa26.Trib1* using three primer sets (table S5). For the atherosclerosis experiments, mice were fed a Western (21% fat and 0.2% cholesterol) diet (829100; Special Diet Services, Braintree, UK) for 12 weeks.

#### *mTrib1 RNA quantification*

BMDMs were cultured for 5 days in complete medium: Dulbecco’s modified Eagle’s medium (DMEM) (BE12-604F, Lonza) containing 10% (v/v) L929-conditioned medium, 10% (v/v) ultralow endotoxin fetal bovine serum (FBS) (S1860-500, Biowest, USA), and penicillin (100 U/ml) and streptomycin (100 ng/ml) (15140-122, Gibco). Total RNA was isolated using a ReliaPrep Kit (Z6011, Promega) and reverse transcribed into cDNA using an iScript cDNA Synthesis Kit (1708890, Bio-Rad). *Trib1* was quantified using the TaqMan assay Mm00454875, which amplifies a 99-nucleotide amplicon comprising exon 2 and 3 sequences.

#### *myeloid-green fluorescent protein (mGFP) quantification*

GFP-positive cells in freshly purified peripheral blood monocytes were isolated by positive selection using magnetic MicroBeads conjugated with F4/80 (130-110-443, Miltenyi Biotec) and CD115 (130-096-354, Miltenyi Biotec) using a modified version of the protocol described by Houthuys *et al.* (*36*). Inflammation was induced by injecting phosphate-buffered saline (PBS) containing thioglycollate into the peritoneal cavity and isolating the infiltrating cells, as described (*37*). The percentages of GFP-positive cells in blood and bone marrow cells (from femurs and tibiae) were determined using mice euthanized humanely by cervical dislocation. Red blood cells (RBCs) were lysed using RBC lysis buffer (00-4300-54, eBioscience). Approximately 10^6^ cells per sample were resuspended in PBS, and dead cells were removed using an amine-reactive dye, NIR Zombie (1:500 dilution in the antibody master mix; 423105, BioLegend). Live cells were stained with the following cell surface marker–specific antibodies, at 0.1 μg/ml each in 100-μl total volume: AF647-conjugated anti-human/mouse CD11b (101220, BioLegend), phycoerythrin (PE)–conjugated anti-mouse Ly6C (101220, BioLegend), and PE/Cy7-conjugated anti-mouse Ly6G (127617, BioLegend). Cells were sorted on an LSR II Cytometer (BD Bioscience) equipped with 355-, 405-, 488-, and 633-nm excitation lasers. Quantifications were performed with FlowJo software.

#### *Blood counts*

Blood counts of heparinized blood, obtained via cardiac puncture, were determined using a Sysmex KX-21 N quantitative automated hematology analyzer.

#### *Histological analysis*

Adipose tissue and liver samples were paraffin-embedded. Cross sections were stained with hemotoxylin and eosin or incubated with F4/80 [1:50; 565409 (clone T45-2342), BD Pharmingen], followed by biotinylated rabbit anti-rat antibody (1:200; Vector Laboratories, UK) and PE-streptavidin (1:20; 405203, BioLegend). They were counterstained with 4′,6-diamidino-2-phenylindole (DAPI) (P36931, Invitrogen) and mounted with ProLong Gold antifade mountant. Mean adipocyte sizes were determined using NIS-Elements software (Nikon Instruments, UK) by measuring at least 15 cells per field of view and three fields of view per mouse. Frozen spleen sections were stained with F4/80 [PE-rat anti-mouse 123019 (clone BM8, BioLegend)] or CD206^−^ [Alexa Fluor 647 rat anti-mouse 321116 (clone 15-2, BioLegend)]–conjugated antibodies (1:200), counterstained with DAPI, and mounted with Aqua-Mount (Thermo Fisher Scientific). Fluorescent images were captured using an inverted wide-field fluorescence microscope (Leica AF6000).

### Models of early-stage human atherosclerosis

For the bone marrow transplantation model, 12- to 13-week-old mixed-gender donor mice were euthanized humanely by cervical dislocation. Femur/tibae bone marrow cells were isolated and purified by standard methods and resuspended in Hank’s balanced salt solution (without phenol red, 14175053, Thermo Fisher Scientific) containing 10% (v/v) fetal calf serum. Donor cells (2 × 10^6^ to 4 × 10^6^) were transplanted via tail vein injection to randomly allocated 12- to 13-week-old male *ApoE*^−/−^ recipient mice that were lethally irradiated with 11 grays (Gy) in two doses (5.5 Gy on two occasions separated by 4 hours) on the day of the transplantation. Bone marrow transplant experiments were undertaken in two waves, one for each m*Trib1* model and respective WT control. During the 7-week posttransplant recovery period, the chimeras were fed a standard chow (2918; Harlan Teklad) diet and then switched to Western diet (21% fat and 0.2% cholesterol, 829100; Special Diet Services, Braintree, UK) for 12 weeks. Chimera mice were given sterile acidified drinking water [1.1% (v/v) HCl] until the end of the procedure.

#### *PCSK9 model*

An adeno-associated virus-based vector (rAAV8) that supports transport of the m*PCSK9^D377Y^* gene to the liver was purchased from UNC GTC Vector Core (Chapel Hill, NC). The mice received 6.1 × 10^11^ viral particles via a single tail vein injection. Following 7-day recovery, they were transferred to the Western diet (829100, Special Diet from Braintree, UK) for 12 weeks.

#### *Lipid measurements*

Fasting, plasma total cholesterol, HDL-C, triglycerides, and glucose levels were measured on a Roche Cobas 8000 modular analyzer. LDL-C was estimated by the Friedewald equation. In addition, colorimetric assays (Cholesterol Quantification Kit, MAK043, Sigma-Aldrich; Triglyceride Assay Kit, ab65336, Abcam) were used.

### Atherosclerotic plaque assessment

Mice were perfused through the heart with PBS and then with 10% (w/v) neutral-buffered formalin. The aorta was segmented at the diaphragm and at the top of the aortic arch. Following careful removal of surrounding extraneous fat and connective tissue, it was dissected longitudinally and fixed in 4% (w/v) paraformaldehyde. Dissected aortas were stained with Oil Red O [60% (v/v) in isopropanol] and pinned onto wax. Images were taken with a macroscopic charge-coupled device camera and analyzed by computer-assisted image analysis (NIS-Elements software, Nikon Instruments, UK). Aortic sinus samples were obtained by excising the heart and transecting parallel to the atria. Following fixation in 10% formalin (v/v) buffered saline for at least 24 hours, samples were serially cut (at 7-μm intervals) from the valve leaflets until the beginning of the aorta.

#### *Immunohistochemistry*

Aortic sinus sections were dewaxed and rehydrated. Heat-mediated antigen retrieval was performed with 10 mM sodium citrate and nonspecific staining reduced by incubation in 5% (v/v) goat serum (G9023, Sigma-Aldrich) for 30 min at room temperature. Primary antibodies diluted as appropriate were as follows: rat anti-mouse MAC-3 antibody clone M3/84 (1:100 dilution, BD Pharmingen), rabbit polyclonal NOS2 antibody (1:100; ab15323; Abcam, UK), rabbit anti-mouse YM1 polyclonal antibody (1:100; ab93034; Abcam, UK) and or rabbit polyclonal OLR1 (1:100; ab203246; Abcam, UK). The secondary antibodies were biotinylated rabbit anti-rat secondary antibody (1:200, Vector Laboratories BA-4000), goat anti-rat DyLight 488 goat anti-rat DyLight 488 (GtxRt-003488NHSX, ImmunoReagents Inc.), and goat anti-rabbit DyLight 550 (GtxRb-003-D550NHSX, ImmunoReagents Inc.). Rabbit anti-rat–conjugated biotinylated antibody was visualized with the Vectastain ABC-HRP complex (PK-6100, Vector Laboratories). Sections were counterstained with Carazzi’s hematoxylin. The number and area of foam cells (MAC-3^+^ macrophages containing a vacuolated cytoplasm) were quantified using NIS-Elements software (Nikon, UK). Fluorescent images were captured using an inverted wide-field fluorescence microscope (Leica AF6000). Quantification of YM1, NOS2, and OLR1 in the aortic sinus lesions was confined to macrophage (MAC-3^+^) cells only and analyzed using ImageJ.

#### *Clinical grading*

The atherosclerotic burden of the atheroma in aortic sinus samples was graded according to the Stary system (e.g., 1 = presence of macrophage foam cells, 2 = presence of intracellular lipid accumulation, and 3 = presence of extracellular lipid pools) (*38*) using a light microscope (Zeiss Axiophot, Carl Zeiss, Jena, Germany) and Image Access. Slides were randomized, and the cardiac pathologist was fully blinded to sample origin.

### Western blotting

Twenty micrograms of total protein lysates was size-fractionated on 4 to 12% NuPAGE Bis-Tris Gel (NP0321, Invitrogen). OLR1 was detected by incubation with a rabbit anti-mouse OLR1 antibody (1:500, ab203246; Abcam), followed by HRP-conjugated goat anti-rabbit immunoglobulin G (IgG) (1:1000; P0448, Dako). LDLR was detected by incubation with a rabbit polyclonal antibody (1:1000; 3839-100, BioVision, USA) at 4°C overnight followed by HRP-conjugated goat anti-rabbit IgG (1:1000; P0448, Dako). α-Tubulin was used as a housekeeping control (sc-32293, Santa Cruz Biotechnology). Detection of immunoreactive products was performed using 1:1 ECL reagent (RPN2235, GE Healthcare) and a C-DiGit Blot scanner (Model 3600, LI-COR). Densitometry was performed with Image Studio Lite software (LI-COR).

### Gene expression studies

RNA from BMDM was prepared as described above. RT-qPCR was performed using primer sequences provided in table S5 and either SYBR-Green (PrecisionPLUS, Primer Design, UK) or a TaqMan (Invitrogen) assay (*TRIB1*, Hs00921832; *OLR1* Hs01552593; *SCARB1,* Hs00969821, Thermo Fisher Scientific). Values were normalized to either *Actb* (Mm02619580, Invitrogen) for mouse samples or *GAPDH* for human samples (Hs02786624, Invitrogen). Fold changes were calculated using the ΔΔ*C*t method.

#### *Microarray analyses*

Details of the CTS, which comprises transcriptomic data from isolated monocytes from 758 donors and matched MDM samples from 596 donors, have been published (*24*). In brief, monocytes were isolated from whole blood using CD14 immunobeads and cultured for 7 days with macrophage colony-stimulating factor (MCS-F) to generate macrophages. The top and bottom quartiles of *TRIB1-*expressing samples were defined respectively as *TRIB1*^High^ and *TRIB1*^Low^. Comparable sizes of differentially expressed gene lists (*n* = 1842 monocytes; *n* = 2171, MDM) were obtained by using false discovery rate (FDR)- adjusted *P* values (i.e., *q* values) of <0.01 plus cutoff log_2_ fold changes of >0.071 (up-regulated) and >−0.071 (down-regulated) for the MDM dataset. The Database for Annotation, Visualization and Integrated Discovery version 6.8 was used to identify for enrichment of functionally related Gene Ontology terms.

#### *Gene expression in polarized human macrophages*

Healthy human monocytes were isolated from whole blood by Ficoll-Paque PLUS (17144003, GE Healthcare) density centrifugation followed by magnetic selection with CD14 Human MicroBeads (130-050-201, Miltenyi Biotec). Monocytes were incubated for 7 days with RPMI 1640 (Gibco) supplemented with MCS-F (100 ng/ml) (300-25-100, PeproTech, UK), 10% FBS (Biowest), 1 mM glutamine (Invitrogen), and 1% penicillin/streptomycin (Gibco), followed by polarization with either LPS (100 ng/ml) (581-007-L002, Enzo Life Sciences, USA) and IFN-γ (20 ng/ml) (300-02, PeproTech, UK), IL-4 (20 ng/ml) (200-04, PeproTech, UK), or IL-10 (20 ng/ml) (200-10, PeproTech, UK) for 24 hours. RNA and RT-qPCR were performed, as described above using the primer sets listed in table S4.

### OxLDL uptake and HDL-mediated cholesterol efflux

Assays were performed on BMDMs cultured for 5 days as described above. OLR1 was detected as described above. For the uptake assays, cells were incubated at 37°C for a further 24 hours with human oxLDL (5685-3557, Bio-Rad) at a concentration of 0 and 25 μg/ml. Total cell lipids were extracted using 7:11:01 (v/v) chloroform: isopropanol:IGEPAL CA-630 (I8896, Sigma-Aldrich), dried at 50°C, and resuspended in 120 μl of cholesterol assay buffer (MAK043, Sigma-Aldrich). Total cholesterol, free cholesterol, and cholesteryl esters were measured using a cholesterol quantification kit (MAK043, Sigma-Aldrich), according to the manufacturer’s instructions. Foam cells were assessed by Oil Red O [60% (v/v) in isopropanol] staining as described above. For the efflux assays, BMDMs were incubated for 24 hours in DMEM (BE12-604F, Lonza) supplemented with 0.2% (w/v) fatty acid–free bovine serum albumin (BSA) (A8806, Sigma-Aldrich) and 2.5 μM TopFluor (Bodipy) cholesterol (Avanti Polar Lipids Inc. USA). The medium was removed, and the cells were washed with PBS and equilibrated for 18 hours in DMEM supplemented with 0.2% (w/v) fatty acid–free BSA. Efflux was measured after a 4-hour incubation period with human HDL (0 and 50 μg/ml) (5685-2004, Bio-Rad). Supernatants were collected, and the cells were lysed with 1% (w/v) cholic acid (C1129, Sigma-Aldrich) in ethanol. Cholesterol efflux was calculated as the percentage of fluorescence (excitation, 490 nm; emission, 520 nm) in the cell medium at the end of the incubation period divided by the total fluorescence in the medium and cells.

### Statistics

All data are reported as means ± SEM unless stated otherwise in the figure legend. Graphs were produced and analyzed by GraphPad Prism software. Each data point represents a single mouse or human donor. For analysis of two groups, Student’s *t* test was performed. When three or more groups were compared, analysis of variance (ANOVA) was used, accompanied with the appropriate ad hoc posttest, as detailed in the specific figure legends. *P* values <0.05 were considered significant.

## Supplementary Material

http://advances.sciencemag.org/cgi/content/full/5/10/eaax9183/DC1

Download PDF

Myeloid Tribbles 1 induces early atherosclerosis via enhanced foam cell expansion

## SUPPLEMENTARY MATERIALS

Supplementary material for this article is available at http://advances.sciencemag.org/cgi/content/full/5/10/eaax9183/DC1 Fig. S1. Expected and observed numbers of 8-week-old offspring with specified *Trib1* genotypes. Fig. S2. *Trib1*^mKO^ and Trib1^mTg^ mice have normal tissue anatomy and F4/80^+^ macrophage numbers. Fig. S3. Plasma lipid levels of chimera and Pcsk9 mice. Fig. S4. Atherosclerotic burden in m*Trib1*➔*ApoE*^−/−^ mice, clinical grading of lesions, and presence of foam cells. Fig. S5. Reciprocal regulation of OLR1 and SCARB1 RNA levels in polarized MDMs. Table S1. Fold changes and *P* values of genes differentially expressed in both MDMs and monocytes. Table S2. Top-ranking biological processes enriched in differentially expressed gene lists of (1) Human *TRIB1*^High^ versus *TRIB*1^Low^ monocytes and (2) between *TRIB1*^High^ versus *TRIB*1^Low^ MDMs. Table S3. The most significantly altered pathways in *TRIB1*^High^ versus *TRIB1*^Low^ macrophages. Table S4. Linoleic (LiA), oleic (OA), and lauric acid (LA) in vitro polarized human MDMs recapitulate the *Olr1*^High^/*Lpl*^High^/*Scarb1*^Low^/*CD36*^WT^ RNA profile *of Trib1*^mTg^ BMDM. Table S5. Primer sequences.

## Appendix

View/request a protocol for this paper from *Bio-protocol*.
