# Supplementary material for: Myeloid Tribbles 1 induces early atherosclerosis via enhanced foam cell expansion
Source: Sci Adv. 2019 Oct 30;5(10):eaax9183. doi: 10.1126/sciadv.aax9183 (PMC6821468; doi:10.1126/sciadv.aax9183)
Supplement: http://advances.sciencemag.org/cgi/content/full/5/10/eaax9183/DC1 [file supp_5_10_eaax9183__index.html]

Science Advances | Science AdvancesAAASSearchScience AdvancesMenu

## Supplementary Materials

**This PDF file includes:**

- Fig. S1. Expected and observed numbers of 8-week-old offspring with specified *Trib1* genotypes.
- Fig. S2. *Trib1*mKO and Trib1mTg mice have normal tissue anatomy and F4/80+ macrophage numbers.
- Fig. S3. Plasma lipid levels of chimera and Pcsk9 mice.
- Fig. S4. Atherosclerotic burden in m*Trib1*➔*ApoE*−/− mice, clinical grading of lesions, and presence of foam cells.
- Fig. S5. Reciprocal regulation of OLR1 and SCARB1 RNA levels in polarized MDMs.
- Table S1. Fold changes and *P* values of genes differentially expressed in both MDMs and monocytes.
- Table S2. Top-ranking biological processes enriched in differentially expressed gene lists of (1) Human *TRIB1*High versus *TRIB*1Low monocytes and (2) between *TRIB1*High versus *TRIB*1Low MDMs.
- Table S3. The most significantly altered pathways in *TRIB1*High versus *TRIB1*Low macrophages.
- Table S4. Linoleic (LiA), oleic (OA), and lauric acid (LA) in vitro polarized human MDMs recapitulate the *Olr1*High/*Lpl*High/*Scarb1*Low/*CD36*WT RNA profile *of Trib1*mTg BMDM.
- Table S5. Primer sequences.

Download PDF

**Files in this Data Supplement:**

- Adobe PDF - aax9183\_SM.pdf
