# Supplementary material for: Myeloid Tribbles 1 induces early atherosclerosis via enhanced foam cell expansion
Source: Sci Adv. 2019 Oct 30;5(10):eaax9183. doi: 10.1126/sciadv.aax9183 (PMC6821468; doi:10.1126/sciadv.aax9183)
Supplement: Download PDF [file aax9183_SM.pdf]

Science Advances
AAAS

advances.sciencemag.org/cgi/content/full/5/10/eaax9183/DC1

Supplementary Materials for

Myeloid Tribbles 1 induces early atherosclerosis via enhanced foam cell expansion

Jessica M. Johnston, Adrienn Angyal, Robert C. Bauer, Stephen Hamby, S. Kim Suvarna, Kajus Baidžajevas, Zoltan Hegedus, T. Neil Dear, Martin Turner, The Cardiogenics Consortium, Heather L. Wilson, Alison H. Goodall, Daniel J. Rader, Carol C. Shoulders*, Sheila E. Francis, Endre Kiss-Toth*

*Corresponding author. Email: c.shoulders@qmul.ac.uk (C.C.S.); e.kiss-toth@sheffield.ac.uk (E.K.-T.)

Published 30 October 2019, *Sci. Adv.* **5**, eaax9183 (2019)
DOI: 10.1126/sciadv.aax9183

This PDF file includes:

Fig. S1. Expected and observed numbers of 8-week-old offspring with specified *Trib1* genotypes.
Fig. S2. *Trib1*^{mKO} and *Trib1*^{mTg} mice have normal tissue anatomy and F4/80^{+} macrophage numbers.
Fig. S3. Plasma lipid levels of chimera and Pcsk9 mice.
Fig. S4. Atherosclerotic burden in m*Trib1*➔*ApoE*$^{-/-}$ mice, clinical grading of lesions, and presence of foam cells.
Fig. S5. Reciprocal regulation of OLR1 and SCARB1 RNA levels in polarized MDMs.
Table S1. Fold changes and *P* values of genes differentially expressed in both MDMs and monocytes.
Table S2. Top-ranking biological processes enriched in differentially expressed gene lists of (1) Human *TRIB1*^{High} versus *TRIB*1^{Low} monocytes and (2) between *TRIB1*^{High} versus *TRIB*1^{Low} MDMs.
Table S3. The most significantly altered pathways in *TRIB1*^{High} versus *TRIB1*^{Low} macrophages.
Table S4. Linoleic (LiA), oleic (OA), and lauric acid (LA) in vitro polarized human MDMs recapitulate the *Olr1*^{High}/*Lpl*^{High}/*Scarb1*^{Low}/*CD36*^{WT} RNA profile *of Trib1*^{mTg} BMDM.
Table S5. Primer sequences.

Supplementary Materials

The Cardiogenics Consortium

François Cambien[1], Panos Deloukas[2], Jeanette Eardman[3], Alison H Goodall[4,5], Christian Hengstenberg[6], Willem H Ouwehand[2,7] Nilesh J Samani[4,5], Heribert Schunkert[3,8]

[1]INSERM UMRS 937, Pierre and Marie Curie University (UPMC, Paris 6) and Medical School, 91 Bd de l'Hôpital 75013, Paris, France

[2]The Wellcome Trust Sanger Institute, Wellcome Trust Genome Campus, Hinxton, Cambridge CB10 1SA, UK

[3]Medizinische Klinik 2, Universität zu Lübeck, Lübeck Germany;

[4]Department of Cardiovascular Sciences, University of Leicester, Glenfield Hospital, Groby Road, Leicester, LE3 9QP, UK

[5]Leicester NIHR Biomedical Research Unit in Cardiovascular Disease, Glenfield Hospital, Leicester, LE3 9QP, UK

[6]Klinik und Poliklinik für Innere Medizin II, Universität Regensburg, Germany;

[7]Department of Haematology, University of Cambridge, Long Road, Cambridge, CB2 2PT, UK and National Health Service Blood and Transplant, Cambridge Centre, Long Road, Cambridge, CB2 2PT, UK

[8]German Center for Cardiovascular Research, Munich Heart Alliance, D-80636 Munich, Germany.

A “Knockout (KO)- first” *Trib1* null allele on C57BL/6 background

	+/+	+/tm1a	tm1a/tm1a
Observed	14	32	**0.0**
Expected	11.5	23	**11.5**
Chi-squared test	0.0004		

B “Conditional-ready” Trib1 null allele

	+/+	+/tm1c	tm1c/tm1c
Observed	9	17	**10**
Expected	9	18	**9**
Chi-squared test	0.92		

C Conditional-*Trib1* null allele (tm1d) on C57BL/6 background

	tm1c/tm1c	tm1c/tm1d	tm1d/tm1d
Observed	20	54	**7**
Expected	25.25	50.5	**25.25**
Chi-squared test	0.0007		

D “KO- first” *Trib1* null allele on mixed genetic background

	+/+	+/tm1a	tm1a/tm1a
Observed	39	77	**25**
Expected	35.25	71	**35.25**
Chi-squared test	0.14		

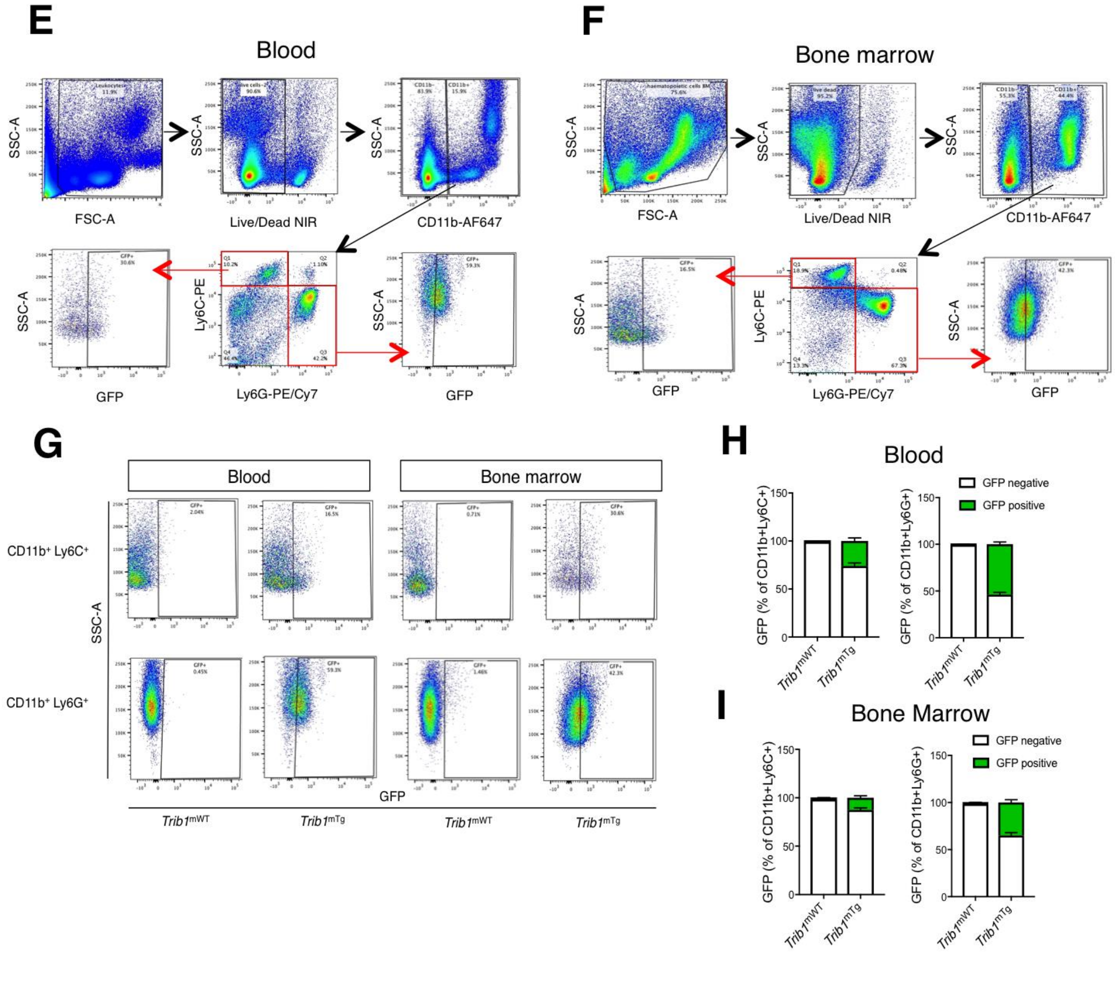

Fig. S1. Expected and observed numbers of 8-week-old offspring with specified *Trib1* genotypes. (**A**) The *Trib1* ‘KO-first’ targeting construct contains elements to produce a

full-body *Trib1* deletion-null allele (tma1). No offspring with a tma1/tma1 genotype were identified from this cross involving two heterozygote parents. (**B**) The expected numbers of mice with each of the three theoretical genotypes (i.e. homozygous WT (+/+), heterozygous (+/tm1c) and homozygous (tm1c/tm1c) from crossing two heterozygote parents were obtained. (**C**) Homozygous conditional-ready (i.e. floxed) *Trib1* null mice (i.e. tm1c/tm1c) were crossed with the heterozygous universal-Cre-recombinase mouse strain B6.Cg-Tg(UBC-cre/ERT2)1Ejb/J. N.B the fewer than expected mice homozygous for Tm1d-null *Trib1* allele. (**D**) Genotype distribution of offspring with the (tma1) *Trib1* deletion null allele on a mixed genetic (C57BL/6 x 129S9) background. (**E**) Representative gating strategy showing SSC-A and FSC-A plots of specified cell populations from blood and bone marrow (**F**) from 10-14 week old *Trib1*^{mWT} and *Trib1*^{mTg} mice (n=4). Live/dead cell discrimination was determined by Zombie NIR amine-reactive dye staining. CD11b^{+} cells were subdivided based on their expression of Ly6C (Y axis) and Ly6G (X-axis). Arrows indicate the fate of specific cell populations. (**G**) TRIB1-GFP expressing cells in CD11b^{+}Ly6C^{+}Ly6G^{-} (top left quadrants of (f)) and CD11b^{+}Ly6C^{-}Ly6G^{+} (bottom right quadrants of (f). (**H**, **I**) Quantification of GFP-positive cells within CD11b^{+}Ly6C^{+}Ly6G^{-} and CD11b^{+}Ly6C^{-}Ly6G^{+} populations of blood and bone marrow cells (n=4, mean ± SEM).

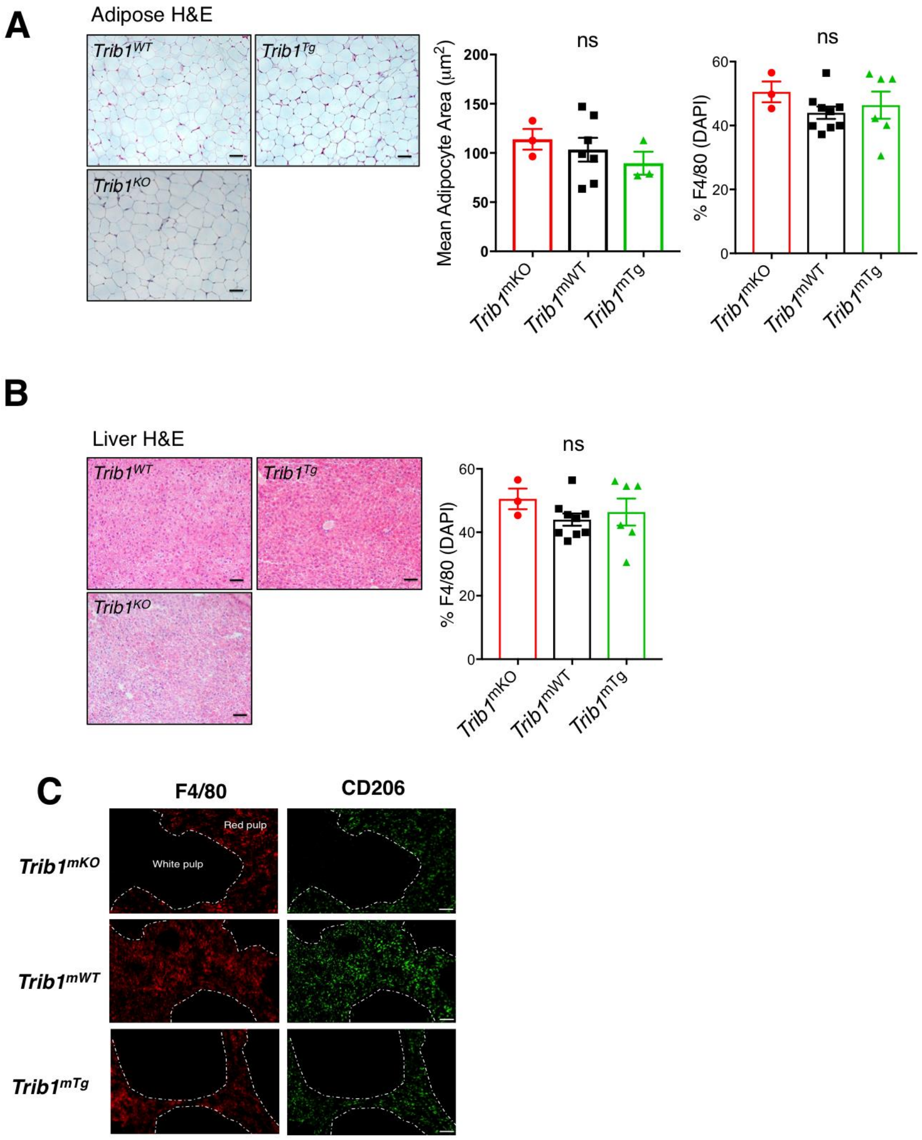

Fig. S2. *Trib1*$^{\mathbf{mKO}}$ and *Trib1*$^{\mathbf{mTg}}$ mice have normal tissue anatomy and F4/80^{+} macrophage numbers. (**A**) Representative H&E staining of adipose tissue (visceral) cross-sections from 10-week old specified mice fed on chow diet. Middle panel, mean adipocyte area of samples (n=3-7, mean ± SEM); Left panel, F4/80+ macrophages contents of adipose tissue samples from specified mice (n=3-7, mean ± SEM). (**B**) Representative H&E staining of liver cross-sections and levels of F4/80+ macrophages in the liver (n=3-9, mean ± SEM); Scale, 20μm. (**C**) Representative IF staining of spleens from *Trib1*^{mKO}, *Trib1*^{mWT}and *Trib1*^{mTg} mice stained with F4/80 (red, left panels) and CD206 (green, right panels). Dotted lines indicate outlines of red and white splenic pulp. Scale: 50μm. ns: non-significant.

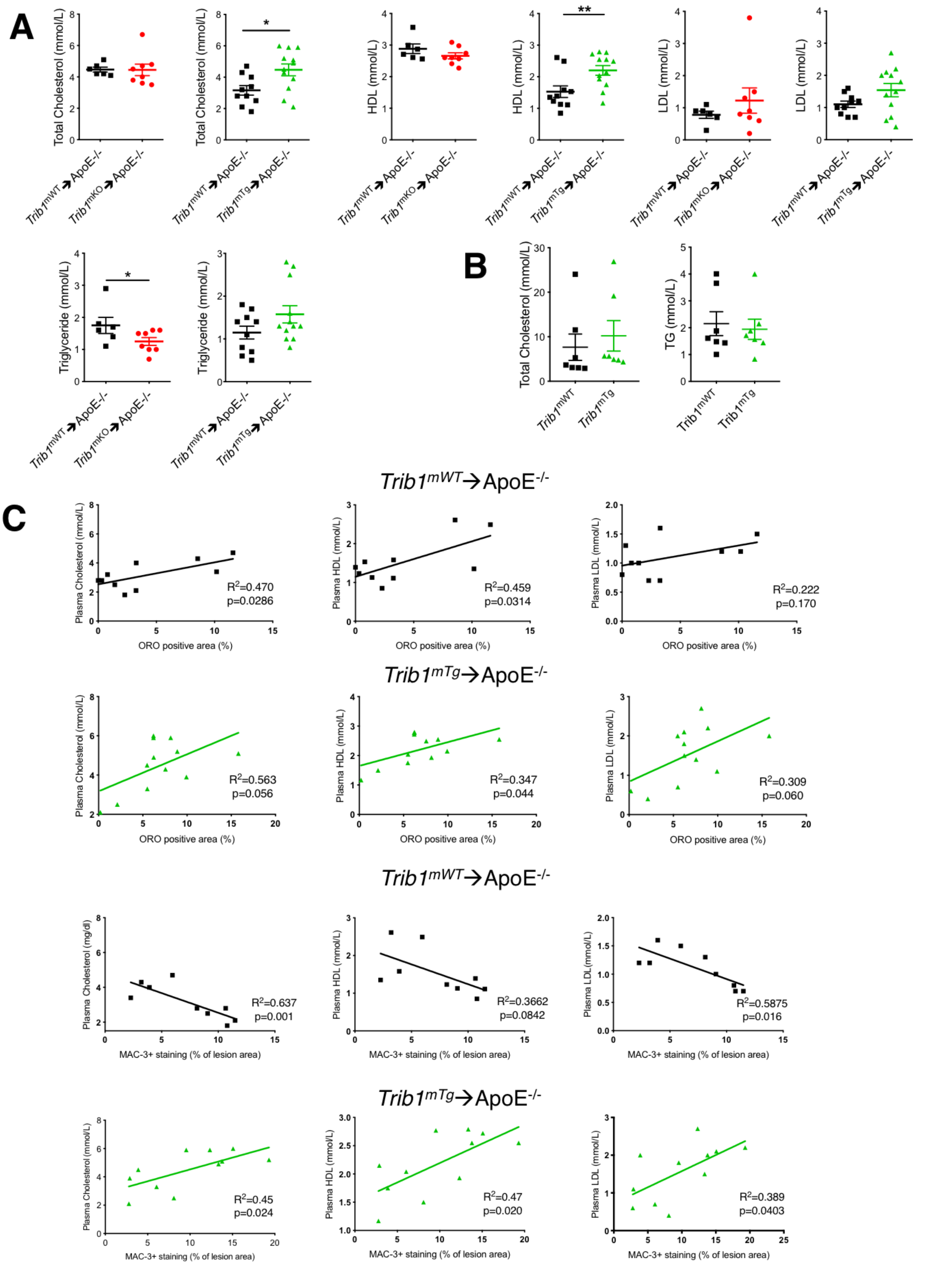

Fig. S3. Plasma lipid levels of chimera and Pcsk9 mice. (**A**) Plasma lipid levels of specified chimera mice following seven weeks recovery and 12 weeks on Western Diet (n=6-12, mean ± SEM). (**B**) Total plasma cholesterol and triglyceride of mTrib1 Pcsk9 mice (n=7, mean ± SEM). (**C**) Correlations in specified mice between total plasma cholesterol (left panel), HDL-C (centre) and LDL-C (right panel) vs. oil Red O (top panels) and MAC-3$^+$ immuno-reactive areas (bottom two panels), expressed as percentage (%) of total lesion area in aortic sinus. Data shows Pearson correlation co-efficient (R^2) along with *P* value (n=9-10).

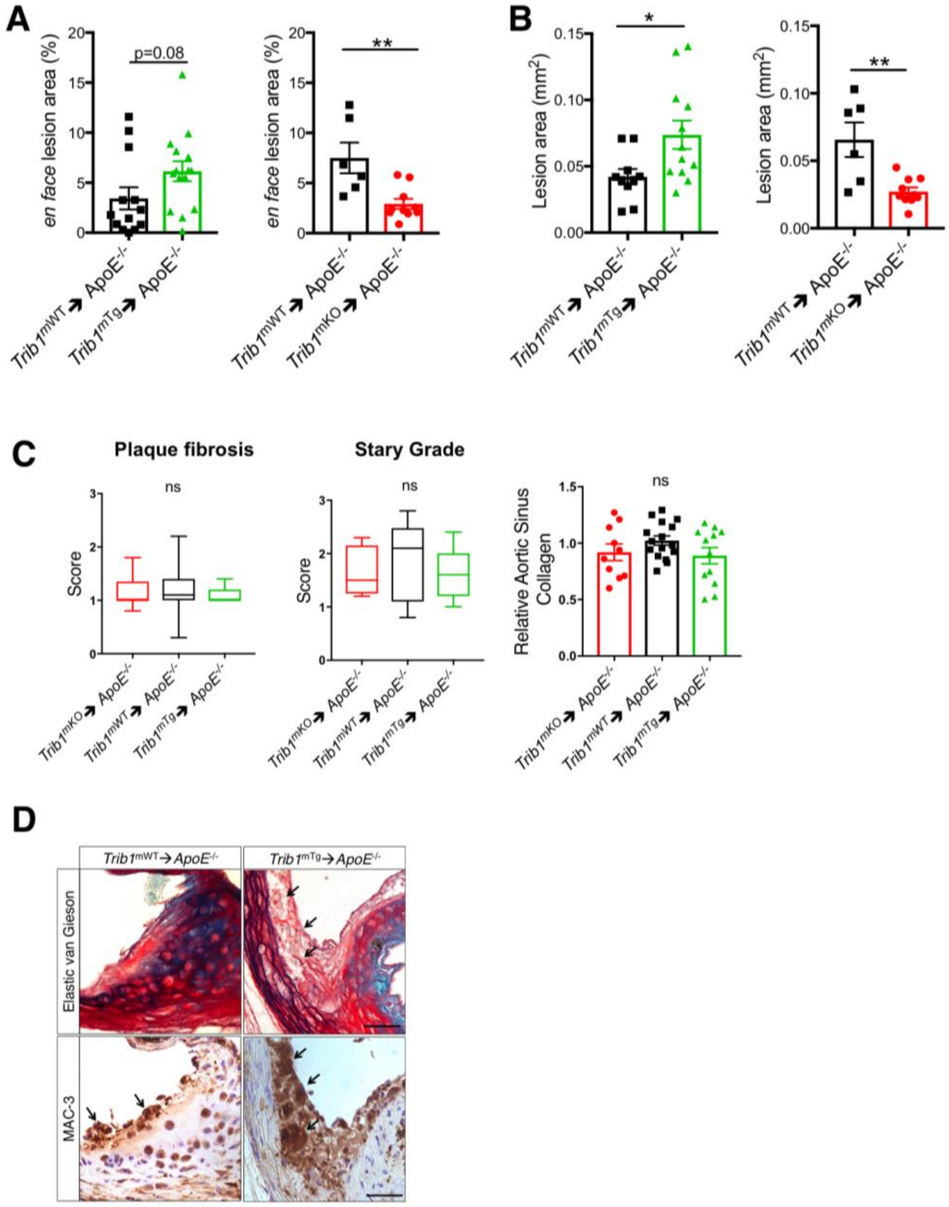

Fig. S4. Atherosclerotic burden in m*Trib1*➔*ApoE*$^{-/-}$ mice, clinical grading of lesions, and presence of foam cells. (**A**) *en face* oil Red O staining of the thoracic aortas (n=6-15, mean ± SEM) and (**B**) lesion sizes in the aortic sinus (n=6-12, mean ± SEM) of *Trib1*^{mWT}→ ApoE$^{-/-}$ versus *Trib1*^{mTg} → ApoE$^{-/-}$ (left-hand panels) and *Trib1*^{mWT}→ ApoE$^{-/-}$ versus *Trib1*^{mKO} → ApoE$^{-/-}$ (right-hand panels) mice. Data are expressed as a percentage (%) of total surface area of the whole aorta. (**C**) Pathological grading of aortic sinus lesions assessing plaque fibrosis (left panel) and overall Stary Grade (e.g. 1 = presence of macrophage foam cells, 2 = presence of intracellular lipid accumulation, 3= presence of extracellular lipid pools) (centre panel) including featured of necrosis and haemorrhage. Multiple lesions per mouse (n=10-16, mean ± minimum and maximum) were scored indicate early stage lesions. Collagen content in the aortic sinus was quantified with Martius Scarlet Blue (right panel, n=10-16, mean ± SEM). (**D**) Representative image of aortic sinus lesion (scale: 30μm) from m*Trib1*→ ApoE$^{-/-}$ mice, with arrows highlight foam cells. Significances were determined by student's t-test (A-B) or one-way ANOVA (C). Ns, non-significant, *P<0.05, **P<0.01.

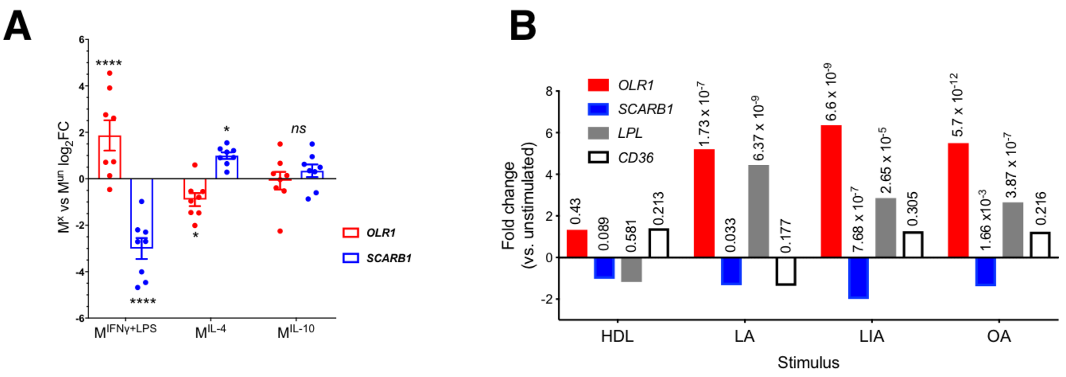

Fig. S5. Reciprocal regulation of OLR1 and SCARB1 RNA levels in polarized MDMs. (**A**) *OLR1* and *SCARB1* RNA levels were quantified by RT-qPCR in human MDMs polarised with interferon γ + lipopolysaccharide (IFN γ + LPS), IL-4 and IL-10. Each data point represents data from one donor (n=8). Log2-fold changes (mean ± SEM) are plotted relative to non-polarised cells**.** (**B**) Fold-changes in levels of specified transcripts in human MDMs polarised with lauric acid (LA), linoleic acid (LIA), oleic and (OA) and HDL. Data were extracted from transcriptome analysis of Xue and colleagues (*39*).

Table S1. Fold changes and *P* values of genes differentially expressed in both MDMs and monocytes.

	Monocyte-derived-Macrophages					Monocytes				
**Gene.ID**	**log.fold**	**fold.change**	**t.statistics**	**p.values**	**q.values**	**log.fold**	**fold.change**	**t.statistics**	**p.values**	**q.values**
AARS	-0.304137224	0.809926432	-8.782897728	1.31E-16	2.69E-14	-0.065066552	0.95590122	-3.843905469	0.000142206	0.001565015
ABHD14A	-0.220750797	0.858118744	-8.037885032	2.20E-14	2.57E-12	-0.098909216	0.9337387	-5.156138259	4.09E-07	1.30E-05
ACAT1	-0.148057487	0.902464766	-5.76394764	2.06E-08	5.23E-07	-0.07291358	0.950716049	-4.575812807	6.46E-06	0.000125567
ADCY3	-0.254850629	0.838073901	-8.218052384	6.54E-15	9.18E-13	-0.158691965	0.895836923	-3.566095551	0.000409236	0.003567832
**ADORA2B**[B]	**-0.38349133**	**0.766580215**	**-8.95060471**	**3.99E-17**	**8.90E-15**	**-0.102855836**	**0.931187866**	**-3.588055639**	**0.000377325**	**0.003367428**
AHR	0.141652554	1.103168031	4.970874874	1.13E-06	1.79E-05	0.16153246	1.118474573	5.087764035	5.74E-07	1.68E-05
AKR7A2	-0.078918283	0.946767257	-3.488746621	0.000559022	0.003634005	-0.072663646	0.950880766	-4.49269597	9.38E-06	0.00017144
ALDH3A2	-0.108851881	0.927325749	-3.553768781	0.00044182	0.002983349	-0.083274197	0.943913003	-4.0872901	5.34E-05	0.000715008
ALG3	-0.085239414	0.942628095	-4.042024821	6.76E-05	0.000611276	-0.052242293	0.964436199	-3.536274173	0.000456637	0.003915552
ALKBH7	-0.101966495	0.931762068	-4.386983346	1.60E-05	0.000180532	-0.077062159	0.94798612	-4.923005676	1.28E-06	3.35E-05
ANAPC11	-0.085872707	0.942214405	-4.44761784	1.23E-05	0.000141461	-0.05873906	0.960102899	-3.585411076	0.00038104	0.003378556
ANKRD37	0.17271609	1.127178572	4.329594849	2.05E-05	0.000222413	0.107501134	1.077360543	4.524879725	8.12E-06	0.000152942
ANO7	0.093401452	1.066882616	4.001031406	7.98E-05	0.000706169	0.101983961	1.073248355	4.397662424	1.43E-05	0.000245118
ANTXR2	0.177347745	1.130803101	6.150376873	2.50E-09	8.29E-08	0.122042637	1.088274604	5.997331838	4.72E-09	2.77E-07
ARHGAP19	0.128541933	1.093188306	5.633869829	4.10E-08	9.67E-07	0.060538818	1.042855174	3.79454163	0.000172403	0.001818891
ARID4A	0.078770827	1.056117847	3.729865936	0.000229583	0.001728025	0.062753195	1.04445707	3.724469238	0.000225804	0.00224404
ARNTL	0.077498448	1.055186819	4.150248844	4.35E-05	0.000424794	0.105821071	1.076106654	5.110912101	5.12E-07	1.54E-05
ATF3	0.192376677	1.142644544	3.71259996	0.000245077	0.001822696	0.402352716	1.321661491	9.92633625	9.00E-21	2.30E-18
ATP6V1F	-0.089599337	0.939783708	-5.656464306	3.64E-08	8.79E-07	-0.089599337	0.961284253	-3.463365081	0.000595061	0.00485921
ATPAF1	-0.073672061	0.950216352	-3.99988055	8.01E-05	0.00070889	-0.064855686	0.956040946	-4.070990188	5.71E-05	0.000758148
BAZ1A	0.074830962	1.053237623	3.207162652	0.001487371	0.008220651	0.093386614	1.066871643	4.589957134	6.06E-06	0.000119581
**BCL3**	**0.239372378**	**1.180479001**	**6.103909899**	**3.24E-09**	**1.04E-07**	**0.099144323**	**1.071137971**	**3.292867123**	**0.001085954**	**0.007829639**
BSCL2	-0.19840344	0.871514492	-5.868162062	1.18E-08	3.20E-07	-0.107471522	0.928213431	-3.947405377	9.43E-05	0.00113083
BTG2	0.317979002	1.246583051	10.29838022	1.85E-21	1.16E-18	0.580118403	1.494971937	15.99920551	2.74E-44	2.14E-41

C10orf59	-0.158281039	0.896092123	-5.486518069	8.81E-08	1.89E-06	-0.072477235	0.951003638	-3.68994754	0.000257517	0.002476517
C12orf10	-0.086294318	0.941939093	-4.979681707	1.08E-06	1.74E-05	-0.07205218	0.951283869	-4.289748921	2.28E-05	0.000360624
C12orf52	-0.086294318	0.941939093	-4.979681707	1.08E-06	1.74E-05	-0.07205218	0.951283869	-4.289748921	2.28E-05	0.000360624
C13orf18	0.440171348	1.35676546	8.548328088	6.77E-16	1.16E-13	0.187277971	1.13861339	4.8072306	2.22E-06	5.18E-05
C16orf7	0.174663087	1.128700788	8.090312857	1.55E-14	1.90E-12	0.09825619	1.070478774	4.426983893	1.25E-05	0.000219117
C17orf101	-0.130454337	0.913543709	-5.174329622	4.23E-07	7.47E-06	-0.057615887	0.960850654	-3.309233856	0.001026123	0.00748459
C3orf62	0.129900536	1.094218259	5.19385354	3.84E-07	6.90E-06	0.088406916	1.063195509	4.597797245	5.85E-06	0.00011629
C5orf5	0.157784854	1.115572947	6.439594797	4.83E-10	1.92E-08	0.083170164	1.059343276	5.789485938	1.49E-08	7.56E-07
C5orf54	-0.112721816	0.924841591	-4.320203558	2.13E-05	0.000229811	-0.089628436	0.939764753	-4.20260421	3.30E-05	0.000485327
C7orf43	0.161297182	1.118292185	6.292869891	1.12E-09	4.00E-08	0.101608485	1.072969067	5.209504439	3.13E-07	1.02E-05
C7orf50	-0.235472322	0.849406868	-6.936902928	2.53E-11	1.48E-09	-0.145898378	0.903816389	-6.128250632	2.25E-09	1.44E-07
C8orf55	-0.11191407	0.925359543	-3.556928637	0.000436758	0.002956334	-0.092692092	0.937771219	-4.065064582	5.85E-05	0.000772729
CALN1	-0.080819265	0.94552056	-4.500936477	9.74E-06	0.000115383	-0.054749069	0.962761884	-3.434654998	0.000659637	0.005242707
CAPG	-0.16007424	0.894979015	-5.987681627	6.15E-09	1.82E-07	-0.087814056	0.940947375	-3.291216034	0.001092166	0.007865358
**CCL3**	**0.38427235**	**1.305201319**	**7.108286426**	**8.83E-12**	**5.78E-10**	**1.350292888**	**2.549638815**	**18.66363469**	**1.89E-55**	**3.94E-52**
**CCL3L1**	**0.396937255**	**1.316709655**	**6.695411776**	**1.08E-10**	**5.17E-09**	**1.183835398**	**2.271799317**	**16.59021024**	**9.55E-47**	**8.53E-44**
**CCL3L3**	**0.365258445**	**1.288112362**	**6.653247048**	**1.39E-10**	**6.42E-09**	**1.356130877**	**2.559977051**	**18.18283748**	**2.01E-53**	**3.58E-50**
**CCL4L1**	**0.457586749**	**1.373242821**	**4.221349367**	**3.23E-05**	**0.000328203**	**0.611835011**	**1.528201743**	**8.981062111**	**1.32E-17**	**2.47E-15**
**CCL8**	**0.443291864**	**1.359703288**	**4.679551789**	**4.38E-06**	**5.80E-05**	**0.30255706**	**1.233328452**	**5.075349313**	**6.10E-07**	**1.76E-05**
CCNG1	-0.149122761	0.901798639	-6.072737251	3.85E-09	1.20E-07	-0.089554517	0.939812905	-4.530583694	7.92E-06	0.00014953
**CD44**	**0.139235411**	**1.101321291**	**3.642123264**	**0.000319136**	**0.002274711**	**0.201566466**	**1.149946279**	**6.311093458**	**7.82E-10**	**5.53E-08**
CDK4	-0.199216252	0.871023621	-12.63759225	1.43E-29	2.99E-26	-0.070434918	0.952350857	-4.043999928	6.38E-05	0.000827016
CEBPD	0.305118352	1.235519989	6.297963502	1.09E-09	3.92E-08	0.100837452	1.072395784	4.908522132	1.37E-06	3.56E-05
CHD4	0.101034254	1.072542082	6.14094502	2.64E-09	8.65E-08	0.050802633	1.035841046	3.737161807	0.000215099	0.002161709
CHMP4A	-0.085559725	0.942418833	-4.873877062	1.79E-06	2.65E-05	-0.085559725	0.942418833	-4.873877062	1.79E-06	2.65E-05
CHSY1	0.103633518	1.074476193	5.740719898	2.33E-08	5.89E-07	0.103633518	1.074476193	5.740719898	2.33E-08	5.89E-07
CKLF	0.074151743	1.052741877	3.477736813	0.000581543	0.003753458	0.050100111	1.035336765	3.260562118	0.001213668	0.008541134

COMMD1	-0.082609651	0.944347896	-4.604258254	6.15E-06	7.78E-05	-0.047869247	0.967363999	-3.71226477	0.000236569	0.002332486
COPE	-0.075498487	0.949014157	-4.5646801	7.34E-06	9.05E-05	-0.065378476	0.955694568	-4.605004898	5.66E-06	0.000113709
CSNK1D	0.108421214	1.078047849	4.826411032	2.23E-06	3.22E-05	0.065883588	1.046725817	3.490105454	0.000540274	0.004500007
CUTA	-0.108198671	0.927745709	-5.822249075	1.51E-08	3.98E-07	-0.057230143	0.961107598	-3.528101273	0.000470499	0.004017879
**CXCL1**	**0.208085468**	**1.155154216**	**3.718390564**	**0.000239774**	**0.001786445**	**0.198932598**	**1.147848786**	**5.388836063**	**1.26E-07**	**4.79E-06**
CYB5R1	-0.098718605	0.933862075	-3.406825538	0.000748195	0.004646763	-0.093376272	0.937326599	-4.823836772	2.05E-06	4.87E-05
DDB1	-0.125380561	0.916762183	-6.437434951	4.89E-10	1.94E-08	-0.059148032	0.95983077	-4.002319239	7.56E-05	0.000937202
DDOST	-0.101472284	0.932081308	-6.219947453	1.69E-09	5.86E-08	-0.057537999	0.960902529	-3.678439906	0.000268988	0.00256318
DKFZP586I1420	0.127170188	1.092149374	3.999501173	8.02E-05	0.000708965	0.061911947	1.043848215	3.211191243	0.001435988	0.009815595
DLG4	0.185386394	1.137121485	5.193134341	3.85E-07	6.91E-06	0.125289074	1.090726261	5.017737027	8.09E-07	2.25E-05
DPH5	-0.080067353	0.946013481	-3.677591437	0.000279567	0.002037985	-0.106135083	0.929073679	-5.056742829	6.69E-07	1.91E-05
DPP7	-0.18572505	0.879207105	-5.217748514	3.41E-07	6.20E-06	-0.086494053	0.941808695	-3.248800407	0.001263528	0.008824934
DUSP2	0.251213063	1.190207458	4.527844672	8.65E-06	0.000104142	0.267930924	1.204079729	8.267138396	2.41E-15	3.83E-13
DUSP5	0.345577117	1.270659179	5.615939962	4.50E-08	1.05E-06	0.248670417	1.18811165	6.113623253	2.45E-09	1.55E-07
DUSP6	0.617173415	1.533867023	10.84644877	2.66E-23	2.56E-20	0.127606397	1.092479643	4.585820364	6.17E-06	0.000121206
DYRK4	-0.083253511	0.943926537	-3.277634972	0.00117149	0.006764882	-0.065051267	0.955911348	-3.570305093	0.000402929	0.003525139
ECE2	-0.096879762	0.935053124	-3.192732554	0.001561088	0.008518869	-0.082940526	0.94413134	-3.565808922	0.000409669	0.003569116
EGR1	0.599880182	1.51559069	9.430304225	1.24E-18	4.43E-16	1.258557259	2.392563572	19.80372323	2.96E-60	9.25E-57
EGR2	0.202074918	1.150351628	4.861143979	1.90E-06	2.79E-05	1.419755997	2.675402581	20.29344534	2.56E-62	1.07E-58
EI24	-0.079329175	0.946497648	-4.519105676	8.99E-06	0.000107518	-0.051303562	0.965063941	-3.457970981	0.000606724	0.004919106
ENOPH1	-0.074301387	0.949801943	-4.475165863	1.09E-05	0.000127401	-0.059563646	0.9595543	-4.272964366	2.45E-05	0.000380763
EPRS	-0.15026855	0.901082715	-5.932946066	8.30E-09	2.39E-07	-0.061128713	0.958513919	-4.263150673	2.55E-05	0.000393711
ESYT1	-0.190315618	0.876413968	-9.160527393	8.84E-18	2.63E-15	-0.072486285	0.950997672	-4.392510335	1.46E-05	0.000250022
ETFB	-0.072510777	0.950981528		0.001029572	0.00608304	-0.093150543	0.937473267		1.81E-07	6.53E-06
EXOSC8	-0.083520291	0.943752005	-3.929664884	0.000105948	0.000896631	-0.081954207	0.944777029	-4.589360716	6.07E-06	0.000119581
FAHD1	-0.07766145	0.947592411	-3.285440488	0.001140629	0.006635713	-0.095970654	0.93564253	-4.476082801	1.01E-05	0.000183254
FAM100B	0.212890808	1.159008228	7.558018214	5.16E-13	4.48E-11	0.137711156	1.100158323	5.632013658	3.50E-08	1.60E-06

FAM118B	-0.039101255	0.973261064	-2.099472163	0.036621846	0.103678967	-0.074048542	0.949968419	-3.367241862	0.000837856	0.00638323
FAM195A	-0.100127511	0.93295053	-3.657724447	0.000301123	0.002167319	-0.105636622	0.929394736	-4.066454887	5.82E-05	0.000770772
FBXO6	-0.13334427	0.911715577	-5.566207073	5.84E-08	1.33E-06	-0.082624987	0.944337858	-3.373696546	0.000819025	0.006262664
FBXW4	-0.086993261	0.941482863	-4.159134352	4.19E-05	0.000412415	-0.06923469	0.95314348	-3.66070969	0.000287609	0.002703522
FCGR2A	0.32179637	1.249885874	5.160308831	4.53E-07	7.90E-06	0.117075212	1.084533947	4.202750242	3.30E-05	0.000485327
FGD4	0.114919685	1.082914758	3.898180416	0.000119935	0.000995637	0.155052231	1.113461929	8.36898061	1.16E-15	1.94E-13
**FOS**	**0.607372682**	**1.52348224**	**12.69986367**	**8.57E-30**	**2.14E-26**	**0.673150675**	**1.59455148**	**18.78229875**	**5.99E-56**	**1.50E-52**
FOSB	0.139458541	1.101491637	3.227001728	0.001391276	0.007765888	1.072955623	2.103738847	16.79284667	1.36E-47	1.31E-44
**FPR2**	**0.241812782**	**1.18247754**	**3.634792169**	**0.000327947**	**0.002325577**	**0.137361934**	**1.099892049**	**3.936734577**	**9.85E-05**	**0.001174557**
FTSJ2	-0.076121105	0.948604682	-3.340020497	0.000945058	0.005664003	-0.083432251	0.943809598	-4.214313827	3.14E-05	0.000464933
FUCA1	-0.210440731	0.864273163	-3.672468488	0.000284982	0.002072625	-0.139875517	0.907597464	-4.562902241	6.85E-06	0.000131676
FUT4	0.083736683	1.059759341	4.226694855	3.16E-05	0.000322511	0.115221443	1.083141287	5.858180876	1.02E-08	5.43E-07
GABARAPL1	0.116479255	1.084086034	5.081546127	6.64E-07	1.12E-05	0.209678591	1.156430522	7.69540838	1.26E-13	1.64E-11
GALIG	-0.286607221	0.819827778	-6.488811919	3.63E-10	1.50E-08	-0.079678483	0.946268507	-3.329186033	0.000957329	0.007077784
GALK1	-0.12262555	0.918514531	-6.161118128	2.35E-09	7.89E-08	-0.044274495	0.969777377	-3.210798353	0.0014379	0.00982329
GNPDA1	-0.080019859	0.946044624	-3.310231335	0.00104755	0.006177582	-0.07734392	0.947800995	-4.305833639	2.13E-05	0.000340155
GSTP1	-0.146655732	0.903342047	-6.930011316	2.63E-11	1.53E-09	-0.076741426	0.948196895	-4.838159566	1.92E-06	4.62E-05
GTPBP6	-0.095082032	0.936219012	-4.005697416	7.83E-05	0.000697101	-0.09003803	0.939497983	-4.428348747	1.25E-05	0.000218109
H3F3B	0.272530861	1.207924984	9.179393049	7.71E-18	2.41E-15	0.231557338	1.174101667	13.2209369	5.06E-33	2.34E-30
**HBEGF**	**0.76361341**	**1.697737499**	**10.26440275**	**2.40E-21**	**1.43E-18**	**0.546514932**	**1.460553224**	**12.21082218**	**4.22E-29**	**1.51E-26**
HDDC3	-0.08217459	0.944632718	-3.739321844	0.000221494	0.001677239	-0.082304525	0.944547644	-4.780695413	2.51E-06	5.72E-05
HDHD2	-0.090442368	0.939234711	-4.118348867	4.96E-05	0.000471872	-0.071304686	0.951776879	-3.717504091	0.00023189	0.00229904
HEATR1	-0.073536338	0.950305749	-3.514536055	0.000509426	0.00335379	-0.107852143	0.927968576	-4.336499811	1.86E-05	0.000307164
HEBP1	-0.240535578	0.84643103	-8.917098822	5.06E-17	1.09E-14	-0.066761639	0.954778749	-4.30180895	2.16E-05	0.000345477
HECA	0.107416644	1.07729745	3.952383836	9.68E-05	0.000831447	0.067760897	1.048088757	3.574215195	0.000397153	0.003484356
HMGN2	-0.078167397	0.947260153	-3.284264147	0.001145231	0.00664702	-0.063817873	0.956728929	-3.248606943	0.001264364	0.008825841
IARS	-0.159480841	0.895347207	-3.828475122	0.000157377	0.001248428	-0.031125924	0.978656226	-2.011458458	0.044992061	0.135409424

IDH1	-0.140574103	0.907158091	-6.634017257	1.55E-10	7.08E-09	-0.057706131	0.960790551	-3.782111794	0.00018091	0.001888821
IER2	0.13043528	1.094623913	5.14783564	4.81E-07	8.38E-06	0.549308043	1.463383647	14.72511368	4.73E-39	2.82E-36
IGFBP6	0.369741009	1.29212085	6.313516874	9.96E-10	3.62E-08	0.084196228	1.060096963	3.815459833	0.000158934	0.001707035
IIP45	0.10876382	1.078303891	3.18207532	0.001617687	0.008793184	0.088757659	1.063454021	3.533027491	0.000462097	0.003959656
**IL1R2**	**0.535136879**	**1.449079633**	**6.47478239**	**3.94E-10**	**1.61E-08**	**0.371667432**	**1.293847364**	**5.229013227**	**2.84E-07**	**9.49E-06**
**IL1RN**	**0.439358621**	**1.356001356**	**5.922585133**	**8.78E-09**	**2.51E-07**	**0.080060824**	**1.057062605**	**3.58684789**	**0.000379017**	**0.003367428**
IMP4	-0.105262596	0.929635717	-4.660880202	4.77E-06	6.22E-05	-0.061355292	0.958363394	-3.423122372	0.000687373	0.005428638
INSIG1	0.193337718	1.143405962	3.713048242	0.000244662	0.001821781	0.169977104	1.12504063	4.859926899	1.73E-06	4.25E-05
INTS3	0.103182485	1.07414033	5.160299818	4.53E-07	7.90E-06	0.086771678	1.061991102	5.234630776	2.76E-07	9.27E-06
IQSEC1	0.12637329	1.091546273	5.633176387	4.12E-08	9.69E-07	0.062226696	1.044075974	3.914810181	0.00010746	0.001260318
ITPRIP	0.150137503	1.10967523	5.616852915	4.48E-08	1.05E-06	0.12930644	1.093767758	6.8158206	3.75E-11	3.76E-09
JUNB	0.303513913	1.234146716	8.533676014	7.49E-16	1.27E-13	0.528021491	1.441950351	14.33023008	1.89E-37	1.03E-34
JUND	0.091852088	1.065737466	4.325519724	2.08E-05	0.000225438	0.190486606	1.141148549	10.46591196	1.16E-22	3.31E-20
**KDM6B**	**0.134598218**	**1.097787044**	**3.779738623**	**0.000189856**	**0.001467891**	**0.135263623**	**1.098293486**	**4.643552159**	**4.74E-06**	**9.87E-05**
KIAA0247	0.150819664	1.11020005	6.861205738	4.00E-11	2.21E-09	0.067099528	1.047608396	3.713980573	0.000235027	0.002322776
KLK7	0.097379743	1.069828649	4.375426099	1.68E-05	0.000187718	0.109934104	1.079178943	5.125490873	4.76E-07	1.46E-05
LCTL	-0.073382755	0.95040692	-3.524884704	0.000490709	0.003251231	-0.077897206	0.947437574	-3.830133355	0.000150085	0.00162456
LGALS3	-0.302035982	0.811106925	-8.011439364	2.63E-14	3.01E-12	-0.095920296	0.935675189	-3.766314934	0.000192294	0.001973778
LHPP	-0.187064511	0.87839119	-4.410614108	1.44E-05	0.000164483	-0.123066078	0.918234104	-5.12094427	4.87E-07	1.48E-05
**LILRB2**	**0.281246918**	**1.215244764**	**5.874882157**	**1.14E-08**	**3.10E-07**	**0.07711268**	**1.054904706**	**4.273221879**	**2.45E-05**	**0.000380763**
LRRC41	-0.111440257	0.925663501	-3.729580825	0.000229831	0.001728852	-0.084673072	0.942998204	-3.586684787	0.000379246	0.003367428
LRRC42	-0.081547692	0.945043281	-4.421582938	1.38E-05	0.000157415	-0.052551539	0.964229491	-3.385452136	0.000785739	0.006048832
LRRC8C	0.15506471	1.11347156	4.456437389	1.18E-05	0.000136621	0.148366148	1.108313596	5.092897854	5.60E-07	1.66E-05
LSMD1	-0.08660218	0.941738111	-4.270120473	2.63E-05	0.000274272	-0.065416821	0.955669167	-3.590226823	0.0003743	0.003349675
**LYN**	**0.124278318**	**1.089962363**	**5.19724828**	**3.78E-07**	**6.81E-06**	**0.110701754**	**1.079753321**	**6.094186571**	**2.73E-09**	**1.72E-07**
MACROD1	-0.156854275	0.896978758	-4.67686575	4.43E-06	5.84E-05	-0.07415812	0.949896268	-3.334758741	0.000938896	0.006982795
MAFB	0.144881451	1.105639797	3.313932327	0.001034277	0.006107949	0.21383436	1.159766491	8.72348876	8.92E-17	1.56E-14

MAP1LC3A	0.428650969	1.345974399	8.976695553	3.31E-17	7.80E-15	0.193058166	1.143184425	4.721697731	3.31E-06	7.22E-05
MAP3K7IP2	0.086358869	1.06168727	3.27720179	0.001173225	0.006771774	0.083928212	1.059900042	3.738332282	0.000214136	0.002159982
MAPKAPK2	0.088045712	1.062929353	3.230745927	0.001373798	0.007690295	0.096529561	1.069198383	4.088519704	5.31E-05	0.000712153
MCL1	0.113188597	1.081616149	4.497360535	9.89E-06	0.000116992	0.242797132	1.18328462	10.53243128	6.75E-23	2.01E-20
MCTP2	0.160146787	1.117400822	4.228528075	3.14E-05	0.000320567	0.15025669	1.109766908	3.724563015	0.000225723	0.00224404
MFNG	-0.154402077	0.89850468	-6.075223264	3.80E-09	1.19E-07	-0.063995929	0.956610857	-4.649530344	4.62E-06	9.64E-05
MGAM	0.098685744	1.07079755	3.32953704	0.000980012	0.005845474	0.184650242	1.136541404	4.182544336	3.59E-05	0.000518822
MIDN	0.125505973	1.090890256	5.564850505	5.88E-08	1.33E-06	0.156433573	1.114528549	8.266422367	2.42E-15	3.83E-13
MRPL48	-0.104044308	0.930421082	-3.66205612	0.000296293	0.002141191	-0.076004498	0.948681358	-4.005017281	7.48E-05	0.000929398
MSL3	0.097200767	1.069695937	3.910750445	0.000114153	0.000952697	0.062734517	1.044443548	3.926668085	0.000102499	0.00121349
MUSK	-0.094047769	0.936890425	-4.882702196	1.71E-06	2.57E-05	-0.061788393	0.958075733	-3.416592166	0.000703557	0.005528514
MXD1	0.122354214	1.088509662	3.19508589	0.00154884	0.008470515	0.238570051	1.179822682	6.647594105	1.05E-10	9.27E-09
MYST3	0.10262168	1.073722871	3.367407341	0.000859143	0.005229313	0.133614279	1.097038593	5.215322173	3.04E-07	1.01E-05
NDUFAF2	-0.106635181	0.928751679	-4.894592217	1.62E-06	2.44E-05	-0.065102849	0.955877171	-3.695443793	0.000252201	0.002446978
NDUFS3	-0.110578468	0.926216608	-4.935707217	1.34E-06	2.07E-05	-0.114339926	0.923804881	-8.126974477	6.46E-15	9.85E-13
NENF	-0.156982628	0.896898959	-5.421725918	1.23E-07	2.52E-06	-0.074490091	0.949677717	-3.234726814	0.001325682	0.00916686
**NFKBIZ**	**0.347874164**	**1.272683923**	**7.267250879**	**3.28E-12**	**2.38E-10**	**0.45296965**	**1.368855016**	**17.01467971**	**1.61E-48**	**1.83E-45**
**NFX1**	**-0.089432833**	**0.939892177**	**-5.033396996**	**8.38E-07**	**1.38E-05**	**-0.056539121**	**0.961568059**	**-3.622581058**	**0.000331836**	**0.003048213**
NIT2	-0.101571536	0.932017187	-4.748863094	3.19E-06	4.42E-05	-0.086244685	0.941971499	-4.765017102	2.70E-06	6.07E-05
**NLRP3**	**0.221092397**	**1.165615847**	**4.100077147**	**5.34E-05**	**0.000502789**	**0.076727127**	**1.054622826**	**4.343716761**	**1.81E-05**	**0.000300174**
NMRAL1	-0.085638949	0.942367083	-3.353984697	0.000900298	0.005442712	-0.080977831	0.945416644	-4.640768734	4.81E-06	9.96E-05
NPTN	0.099185878	1.071168824	6.70178539	1.04E-10	5.00E-09	0.043258145	1.030438316	3.398397344	0.000750544	0.005817299
NR2C2AP	-0.100623338	0.932629948	-3.917973783	0.000110951	0.000929187	-0.088165754	0.940718021	-4.161258603	3.93E-05	0.000558092
NSMCE2	-0.152545494	0.899661697	-6.588525854	2.03E-10	8.90E-09	-0.059179036	0.959810143	-4.029542009	6.77E-05	0.000864092
NUBP1	-0.085481669	0.942469823	-4.02009873	7.39E-05	0.000663469	-0.052435844	0.96430682	-4.081918351	5.46E-05	0.000730235
**OSM**	**0.321101239**	**1.24928379**	**5.800203377**	**1.70E-08**	**4.42E-07**	**0.700639974**	**1.625225576**	**15.24872599**	**3.42E-41**	**2.25E-38**
PDCL3	-0.082740008	0.944262572	-3.824633135	0.000159733	0.001265513	-0.050043586	0.965907147	-3.267262309	0.001186087	0.008401392

PDE4B	0.327127017	1.254512649	5.714525947	2.68E-08	6.72E-07	0.160562852	1.117723121	4.065153201	5.85E-05	0.000772729
PDIA5	-0.193318073	0.874591921	-5.466876833	9.74E-08	2.06E-06	-0.095870481	0.935707498	-4.398492832	1.42E-05	0.000244593
PDLIM4	0.178257976	1.131516777	3.871595217	0.000133091	0.001088233	0.068099831	1.048335015	3.27579928	0.001151787	0.008233069
PDS5B	0.079997806	1.057016433	3.381533147	0.000817731	0.005013863	0.06686481	1.047437971	3.764787918	0.00019343	0.001983805
PDXP	-0.118804266	0.920950636	-5.028452585	8.58E-07	1.41E-05	-0.089541741	0.939821228	-4.275920652	2.42E-05	0.000377386
PEMT	-0.137906939	0.908836739	-5.568717429	5.76E-08	1.32E-06	-0.068638674	0.953537331	-3.740529268	0.00021234	0.002149535
PEPD	-0.130715773	0.913378178	-6.822591139	5.05E-11	2.71E-09	-0.048453321	0.966972442	-3.315473055	0.001004132	0.007345616
PHACTR2	0.114677963	1.082733332	4.647679352	5.06E-06	6.57E-05	0.096806678	1.069403778	4.486815565	9.63E-06	0.0001754
PIK3CD	0.075858421	1.053987985	3.304209788	0.001069486	0.006295061	0.063236491	1.044807017	3.651845782	0.000297366	0.002776453
PIK3CG	0.128019611	1.092792593	3.948743786	9.82E-05	0.000840657	0.102561997	1.073678453	3.896477026	0.000115579	0.001336713
PLCB2	0.14419825	1.105116335	4.633572923	5.39E-06	6.94E-05	0.11826077	1.085425547	5.143517986	4.35E-07	1.36E-05
**PLEK**	**0.183221455**	**1.135416376**	**5.253313602**	**2.86E-07**	**5.31E-06**	**0.083788268**	**1.059797234**	**3.292007573**	**0.001089184**	**0.007848401**
PLEKHG2	0.162183288	1.118979254	4.789346908	2.65E-06	3.75E-05	0.088080544	1.062955016	3.909007539	0.00010997	0.001282742
PMAIP1	0.156305945	1.114429957	4.084295661	5.70E-05	0.000529856	0.340874612	1.266524172	8.756108303	7.02E-17	1.25E-14
PNRC1	0.111342396	1.080232902	4.014727395	7.55E-05	0.000675048	0.189766999	1.140579493	8.085652817	8.63E-15	1.28E-12
POFUT2	-0.081974139	0.944763976	-4.229119756	3.13E-05	0.000320035	-0.057976816	0.960610301	-3.34313745	0.000911801	0.006834137
POLR1C	-0.074944427	0.94937869	-3.623946854	0.000341401	0.002407333	-0.094174239	0.936808299	-5.554022368	5.30E-08	2.26E-06
PPA2	-0.07420225	0.949867213	-3.287428391	0.001132891	0.006596835	-0.082983799	0.944103021	-5.08803853	5.73E-07	1.68E-05
PPM1B	0.123715347	1.089537118	3.907006002	0.000115847	0.000964904	0.197387686	1.146620268	6.837722535	3.28E-11	3.33E-09
PPP1R14A	0.101993702	1.073255601	4.513812499	9.20E-06	0.000109745	0.096762875	1.069371309	3.737777955	0.000214592	0.002160668
PRDX4	-0.122274476	0.918738075	-5.354852024	1.72E-07	3.40E-06	-0.063036505	0.957247236	-3.935635899	9.89E-05	0.001178603
PRKCA	0.168047696	1.123537047	4.118823258	4.95E-05	0.000471313	0.117445353	1.084812234	3.731002385	0.000220233	0.00219916
PRPF38B	0.074819111	1.053228971	3.887352861	0.000125137	0.001029932	0.082492927	1.05884611	4.870438567	1.64E-06	4.10E-05
PSCD4	0.16455209	1.120818046	6.458496931	4.33E-10	1.75E-08	0.085713665	1.061212567	3.780685422	0.000181912	0.001890489
PSMG1	-0.163159796	0.893066929	-6.982837056	1.91E-11	1.15E-09	-0.055318577	0.962381906	-3.34189826	0.000915762	0.006855604
**PTGER4**	**0.168539459**	**1.123920086**	**4.900698401**	**1.58E-06**	**2.38E-05**	**0.126426806**	**1.091586764**	**5.544644821**	**5.57E-08**	**2.34E-06**
PTGES2	-0.079042844	0.946685517	-3.526549762	0.000487758	0.003241867	-0.129356795	0.91423896	-7.036804222	9.41E-12	1.01E-09

PTGS2	0.141803996	1.103283838	3.516518304	0.00050579	0.003333361	0.885610294	1.847546011	18.06600062	6.22E-53	9.72E-50
PTPN1	0.076072969	1.054144738	4.045351584	6.67E-05	0.000604506	0.05852511	1.041400576	3.959030897	9.00E-05	0.001088681
RARA	0.204796892	1.152524078	5.642775463	3.91E-08	9.30E-07	0.080018282	1.057031435	4.019030828	7.06E-05	0.000892163
RFX2	0.122455843	1.088586344	3.959551171	9.41E-05	0.000812012	0.225426628	1.169122926	6.070595993	3.12E-09	1.94E-07
RGL2	0.130432094	1.094621497	5.221786219	3.34E-07	6.10E-06	0.122475235	1.088600976	5.925625387	7.05E-09	3.86E-07
RGS2	0.229580862	1.172494261	4.696722507	4.05E-06	5.45E-05	0.168739653	1.124076056	8.334394873	1.49E-15	2.42E-13
RNF113A	-0.082422209	0.944470598	-3.258936807	0.001248599	0.007095446	-0.116217893	0.92260314	-5.092753527	5.60E-07	1.66E-05
RNF149	0.096919046	1.069487074	3.731752536	0.000227947	0.001717781	0.131518445	1.095446058	4.738500744	3.06E-06	6.76E-05
RNF217	0.127029555	1.092042917	5.112944546	5.70E-07	9.74E-06	0.084240061	1.060129172	3.81238199	0.000160851	0.001718771
RNPEP	-0.177302645	0.884354898	-4.931097549	1.37E-06	2.10E-05	-0.11881042	0.920946708	-3.65914325	0.000289311	0.002715439
RNU2-1	0.089136211	1.0637331	3.154657464	0.001772137	0.009435798	0.232229619	1.174648913	4.8201538	2.09E-06	4.92E-05
RPP21	-0.094224611	0.93677559	-4.38143606	1.64E-05	0.000183906	-0.056630747	0.961506991	-3.770796441	0.000188998	0.001951156
RPUSD3	-0.090199345	0.939392939	-5.022377427	8.84E-07	1.45E-05	-0.057699648	0.960794869	-4.009102015	7.36E-05	0.000916105
S100A6	-0.099892632	0.933102432	-5.116743895	5.60E-07	9.60E-06	-0.053921892	0.963314047	-3.269435636	0.001177266	0.008376881
SARS2	-0.106524186	0.928823136	-4.087708421	5.62E-05	0.000523705	-0.092005007	0.93821794	-3.362818804	0.000850992	0.006467541
SEC11C	-0.119082005	0.920773358	-4.287702423	2.45E-05	0.000257837	-0.100547919	0.932678703	-4.750360269	2.90E-06	6.43E-05
SERPINB6	-0.165039723	0.891903962	-6.598549199	1.91E-10	8.45E-09	-0.072064957	0.951275444	-4.208462677	3.22E-05	0.000476011
SERTAD1	0.084364397	1.060220541	3.98495583	8.51E-05	0.000743052	0.074447725	1.052957878	4.179484188	3.64E-05	0.000524785
SH2B1	0.09856572	1.07070847	3.932551086	0.000104746	0.000889027	0.078067408	1.055603038	3.435970305	0.000656541	0.005225323
SLC18A1	0.098355691	1.070552606	3.791154669	0.000181724	0.001418174	0.0990115	1.071039361	3.338810689	0.000925701	0.006905097
SLC22A4	0.319186567	1.247626902	6.6158757	1.73E-10	7.74E-09	0.096331762	1.069051802	3.837675718	0.00014572	0.0015897
SLC25A25	0.091011409	1.065116626	3.779264106	0.000190201	0.001469652	0.100590778	1.072212439	4.019269151	7.06E-05	0.000892163
SLC25A37	0.192104326	1.142428857	4.991330908	1.03E-06	1.66E-05	0.171914461	1.126552432	4.10071437	5.05E-05	0.000685251
SLC39A11	-0.129511434	0.91414097	-4.785507913	2.70E-06	3.80E-05	-0.071631819	0.951561087	-3.913258903	0.000108125	0.001264532
SLC47A1	-0.438124158	0.73809368	-7.513785862	6.85E-13	5.79E-11	-0.153173451	0.899270189	-3.913505762	0.000108019	0.001264473
SLC7A1	-0.215612978	0.861180181	-5.23197469	3.18E-07	5.86E-06	-0.088604519	0.940431965	-4.202197849	3.31E-05	0.000485327
SMU1	-0.091258964	0.938703235	-3.685538694	0.000271358	0.00198809	-0.093463147	0.937270157	-3.996267862	7.75E-05	0.000954403

SNF8	-0.109213554	0.927093304	-3.561845788	0.000428989	0.002913213	-0.090036648	0.939498883	-3.594467712	0.000368457	0.003316378
SNTA1	-0.101696995	0.93193614	-4.957948309	1.20E-06	1.89E-05	-0.079569109	0.946340249	-4.281244365	2.36E-05	0.000371664
SNTB1	-0.211121373	0.863865508	-6.269608528	1.28E-09	4.51E-08	-0.07764309	0.947604471	-3.247651786	0.001268498	0.008845789
SON	0.075373826	1.053634015	3.141570888	0.001850561	0.009790825	0.254330085	1.192781747	11.63066244	6.57E-27	2.16E-24
SORL1	0.217433749	1.162663612	4.243383738	2.95E-05	0.000302432	0.155869881	1.114093164	5.317985172	1.81E-07	6.53E-06
**SP100**	**0.074333565**	**1.052874561**	**3.147790262**	**0.001812899**	**0.009628234**	**0.10708263**	**1.077048062**	**4.86147341**	**1.72E-06**	**4.23E-05**
SPATA2L	0.077883117	1.055468203	3.306623063	0.001060644	0.006248902	0.082802203	1.059073123	3.657107	0.000291538	0.00273019
SRGN	0.120913519	1.087423204	3.566045338	0.000422457	0.002875101	0.091791169	1.065692465	5.033841392	7.48E-07	2.12E-05
SSH1	0.117886582	1.08514406	3.275325526	0.001180769	0.006801375	0.092150307	1.065957787	3.528921079	0.000469091	0.004011335
SSR4	-0.154674864	0.898334805	-7.311714215	2.48E-12	1.87E-10	-0.081743484	0.944915035	-5.285984039	2.13E-07	7.53E-06
STRA13	-0.102395792	0.931484849	-4.550799609	7.81E-06	9.54E-05	-0.068303416	0.953758943	-4.469907997	1.04E-05	0.000186722
STX8	-0.106395069	0.928906267	-4.97611805	1.10E-06	1.76E-05	-0.088080028	0.94077392	-4.768060974	2.67E-06	6.01E-05
TCEB2	-0.12807382	0.915052346	-7.164054602	6.25E-12	4.24E-10	-0.053410397	0.963655642	-3.897313069	0.000115197	0.001335985
TGFA	0.207188734	1.154436432	4.902585253	1.56E-06	2.36E-05	0.25810278	1.195904992	7.682987411	1.37E-13	1.77E-11
THOC6	-0.102747106	0.931258048	-4.935452565	1.34E-06	2.07E-05	-0.050463218	0.965626238	-3.313130403	0.001012337	0.007396983
TLE4	0.099582303	1.071463202	3.962745287	9.29E-05	0.000803427	0.095692412	1.068578142	6.151186144	1.97E-09	1.28E-07
**TLR1**	**0.212698796**	**1.158853983**	**5.920077987**	**8.90E-09**	**2.54E-07**	**0.144246725**	**1.105153468**	**5.979914611**	**5.21E-09**	**3.01E-07**
**TLR6**	**0.118636299**	**1.085708117**	**5.635337649**	**4.07E-08**	**9.62E-07**	**0.191448304**	**1.141909489**	**9.762034106**	**3.30E-20**	**8.08E-18**
TMED3	-0.156932298	0.89693025	-6.041643793	4.57E-09	1.40E-07	-0.077397534	0.947765773	-3.694631609	0.00025298	0.002449853
TMEM108	-0.083008478	0.944086871	-3.226132034	0.001395365	0.007784405	-0.075176839	0.949225762	-3.406084436	0.000730346	0.00570318
TMEM119	0.407320849	1.326220673	4.852267483	1.98E-06	2.90E-05	0.090267444	1.064567511	3.323504339	0.000976469	0.007198008
TMEM126B	-0.09773921	0.934496257	-4.190760703	3.67E-05	0.000366626	-0.073910422	0.950059371	-3.814685801	0.000159414	0.001709726
TMEM185B	0.087540945	1.062557523	4.512213377	9.26E-06	0.000110417	0.109293468	1.078699835	5.545711283	5.54E-08	2.33E-06
TMEM4	-0.077282717	0.947841204	-4.299799302	2.32E-05	0.000246188	-0.048000547	0.967275963	-3.318931382	0.000992132	0.007283404
TMEM43	0.113626321	1.081944369	5.403639932	1.34E-07	2.74E-06	0.080609124	1.057464421	5.069355037	6.28E-07	1.80E-05
TMEM86B	0.132776113	1.096401428	4.832430923	2.17E-06	3.16E-05	0.099500867	1.071402722	5.01787758	8.08E-07	2.25E-05
TMEM88	0.113122368	1.081566497	3.774864035	0.000193432	0.001488176	0.210288183	1.156919259	7.519166485	4.11E-13	4.98E-11

TMUB1	0.10526693	1.075693399	5.09535617	6.21E-07	1.06E-05	0.061799654	1.04376697	3.308876704	0.001027395	0.007485344
TNFAIP3	0.278513353	1.212944343	8.39573452	1.94E-15	3.11E-13	0.131558543	1.095476505	4.412551789	1.34E-05	0.000231533
TOMM40	-0.156370358	0.897279678	-4.358870061	1.81E-05	0.000199781	-0.067701725	0.9541568	-3.598877343	0.000362472	0.003281412
TP53INP2	0.071232699	1.050613989	4.410001098	1.45E-05	0.000164771	0.087800336	1.062748583	4.915240647	1.33E-06	3.47E-05
TRAPPC2L	-0.102562753	0.931377055	-3.813981748	0.000166441	0.001307883	-0.099801477	0.933161391	-5.347559655	1.55E-07	5.71E-06
TRAPPC6A	-0.139423253	0.907882027	-4.798217477	2.54E-06	3.61E-05	-0.0762047	0.948549719	-3.248868851	0.001263233	0.008824934
TRIB3	-0.505640758	0.70434748	-7.257304883	3.49E-12	2.46E-10	-0.097255745	0.93480947	-3.551400268	0.000431986	0.003732334
TSPAN4	-0.221314221	0.857783684	-4.710113765	3.81E-06	5.18E-05	-0.158522667	0.895942054	-5.253527858	2.51E-07	8.60E-06
TXNRD2	-0.150498275	0.900939244	-5.915606515	9.12E-09	2.58E-07	-0.067631696	0.954203117	-3.461327303	0.000599442	0.004875878
UBE2D4	-0.146220347	0.903614705	-6.845449803	4.40E-11	2.41E-09	-0.066175723	0.955166588	-3.609610983	0.000348285	0.003173654
UBP1	0.091088361	1.06517344	4.827129867	2.22E-06	3.22E-05	0.081511559	1.058126093	4.307660668	2.11E-05	0.000339171
UQCRC2	-0.123570753	0.91791295	-4.830210277	2.19E-06	3.18E-05	-0.060680077	0.958812035	-3.387712162	0.000779485	0.00601551
UROD	-0.131555255	0.912846852	-5.453197036	1.04E-07	2.19E-06	-0.061655979	0.958163672	-3.676231608	0.000271244	0.002572909
**VARS**	**-0.085127761**	**0.94270105**	**-3.400475399**	**0.000765115**	**0.004728356**	**-0.084732456**	**0.942959389**	**-4.659160596**	**4.42E-06**	**9.25E-05**
VNN3	0.161419074	1.118386673	3.457440708	0.000625299	0.003982418	0.2947784	1.226696541	6.390723	4.90E-10	3.74E-08
WASPIP	0.104509289	1.075128639	4.490045346	1.02E-05	0.00012036	0.091601082	1.065552061	4.799488687	2.30E-06	5.35E-05
WBP11	0.090806583	1.064965417	4.702803815	3.94E-06	5.33E-05	0.084585568	1.06038309	4.75980026	2.77E-06	6.20E-05
YARS	-0.157195994	0.896766323	-7.66595984	2.56E-13	2.36E-11	-0.040546757	0.972286397	-3.569282261	0.000404453	0.003533526
YIF1A	-0.107681134	0.928078579	-3.821679167	0.000161567	0.001276809	-0.079763628	0.946212662	-3.640561673	0.000310238	0.002884527
YPEL5	0.075428218	1.053673739	3.846044248	0.000147015	0.001178947	0.103422692	1.074319188	6.630585064	1.17E-10	1.01E-08
ZBTB24	-0.084531568	0.9430907	-4.770365716	2.89E-06	4.05E-05	-0.097970295	0.934346585	-6.759323447	5.32E-11	5.12E-09
ZBTB3	-0.104260947	0.930281378	-4.153533406	4.29E-05	0.000420087	-0.116240583	0.922588629	-5.281923335	2.17E-07	7.67E-06
ZEB2	0.10072671	1.072313469	3.421123906	0.000711368	0.004464619	0.15967798	1.11703778	6.970708709	1.43E-11	1.51E-09
ZMYND15	0.253099048	1.191764392	6.232534497	1.58E-09	5.49E-08	0.131169235	1.095180933	3.740695801	0.000212204	0.002149535
ZSWIM4	0.45012701	1.366160524	7.895154562	5.70E-14	5.99E-12	0.50124646	1.415435943	15.78706282	2.07E-43	1.52E-40

[A] Please see Methods for cut-off values for definition of differential expression. The differentially expressed MDM gene list contained 2171 genes compared to 1842 in the monocytes. Only those that display concordant differential expression *in* $TRIB1^{High}$ versus $TRIB1^{Low}$ cells are shown. [B] Grey highlighting indicates those Genes assigned by DAVID to the GO 0006954 'Inflammatory Response' cluster. There was a 2.84-fold over-representation of these genes in this 'Cluster of Terms but P values (P= 6.96 x 10^{-5}, Benjamini-Hochberg adjusted *P* value 1.058 X 10^{-1}) were modest.

Table S2. Top-ranking biological processes enriched in differentially expressed gene lists of (1) Human *TRIB1*^{High} versus *TRIB*1^{Low} monocytes and (2) between *TRIB1*^{High} versus *TRIB*1^{Low} MDMs.

Monocytes					Monocyte Derived Macrophages			
Cluster description[A]	Rank	Cluster Gene Ontology terms in differentially expressed gene-sets (DEG[B])	Cluster enrichment in DEG of *TRIB1*^{High} monocytes	p value[C] ranges	Rank	Cluster Gene Ontology terms in differentially expressed gene-sets (DEG[B])	Cluster enrichment in DEG of *TRIB1*^{High} MDMs	p value[C] ranges
Ribonucleprotein Complex	1[D]	0022613; 0042254; 0034660; 0016072; 0006364; 0034470	8.78	3.9×10^{-9}- 2.6×10^{-5}	-	-	-	-
Oxoacid, Carboxylic & Fatty acid metabolism	-				1	0043436; 0019752; 0006631	8.28	7.9×10^{-8} - 3.3×10^{-3}
Apoptosis & Cell Death	2	0006915[E]; 0012501; 0016265; 0008219	6.87	6.8×10^{-6} - 1.6×10^{-4}	-		-	-
Signal transduction & Cell communication	-		-	-	2	0009966; 0010648; 0009968; 1902532	7.62	1.1×10^{-10}- 3.4×10^{-4}
Regulation of Cell Death & Apoptosis	3	00043067[E]; 0042981[E]; 0043069[E]; 0060548[E]; 0043066[E]; 0043068[E]; 0010942[E]; 0043065[E]; 0010941; 0006916; 0006917; 0012502	6.03	6.5×10^{-7}- 5.2×10^{-3}	4	0043067; 0042981; 0043069; 0006915; 0060548; 0043066; 0043068; 0010942; 0043065;	6.81	8.4×10^{-8}- 1.48×10^{-3}
Response to unfolded proteins	13	0006986[F]	2.53	8×10^{-3}	3	0006986; 0034620; 0030968; 0036498; 0035967	6.91	9.0×10^{-8}- 1.48×10^{-4}
Macromolecular Complex Subunit Organisation	4	0034621; 0034622; 0043933; 0065003; 0070271	5.72	7.33×10^{-6} - 4.82×10^{-2}	-	-	-	

[A]Annotation was performed using the Database for Annotation, Visualization and Integrated Discovery (DAVID) Platform.

[B]DEG lists, defined as in the Methods Section, comprised 1842 (monocytes) and 2171 (MDM) genes.

[C] The monocyte and MDM DEG lists returned respectively 13 and 27 GO clusters with Benjamini-Hochberg adjusted P values > 5.0×10^{-2}

[D] Benjamini-Hochberg adjusted P values

[E] GO term also present in MDM Cluster 4 of GO terms

[F] GO term also present in MDM Cluster 3 of GO terms

Table S3. The most significantly altered pathways in *TRIB1*^{High} versus *TRIB1*^{Low} macrophages.

Monocyte Derived Macrophages						Monocytes		
Pathway No.	Pathway Name	Rank-ing	Log. fold change	p value	FDR	Log. fold change	p value	FDR
645	Creation of C4 and C2 Activators	1	0.34	1.6×10^{-5}	7.3×10^{-5}	Not present		
199	Translocation of Zap 70 to Immunological Synapse	2	0.20	$< 10^{-7}$	$< 10^{-7}$	-0.03	0.45	0.54
380	PD1 Signaling	3	0.17	$< 10^{-7}$	$< 10^{-7}$	-0.02	0.60	0.67
197	Phosphorylation Of CD3 And TCR Zeta Chains	4	0.16	$< 10^{-7}$	$< 10^{-7}$	-0.03	0.46	0.54
222	**HDL Mediated Lipid Transport**	5	0.16	$< 10^{-7}$	$< 10^{-7}$	0.01	0.36	0.43
263	**Chemokine Receptors Bind Chemokines**	6	0.15	$< 10^{-7}$	$< 10^{-7}$	0.23	$< 10^{-7}$	$< 10^{-7}$
647	Initial Triggering of Complement	7	0.15	5.1×10^{-4}	1.7×10^{-3}	0.01	0.74	0.79
202	Generation of Second Messenger Molecules	8	0.13	$< 10^{-7}$	$< 10^{-7}$	-0.01	0.59	0.67
604	**Lipoprotein Metabolism**	9	0.11	$< 10^{-7}$	$< 10^{-7}$	Not present		
270	Norepinephrine Neurotransmitter Release Cycle	10	0.10	1.9×10^{-6}	9.5×10^{-6}	0.04	1×10^{-3}	2×10^{-3}
280	Steroid Hormones	11	0.09	$< 10^{-7}$	$< 10^{-7}$	-0.01	0.45	0.54
24	Metabolism of Steroid Hormones and Vitamins A and D	12	0.09	$< 10^{-7}$	$< 10^{-7}$	-0.01	0.22	0.29
246	**Peptide Ligand Binding Receptors**	13	0.09	$< 10^{-7}$	$< 10^{-7}$	0.11	$< 10^{-7}$	$< 10^{-7}$
405	Zinc Transporters	14	0.08	2.3×10^{-6}	1.1×10^{-5}	-0.02	0.03	0.05
607	**Chylomicron Mediated Lipid Transport**	15	0.08	6.2×10^{-4}	2.0×10^{-3}	-4.35×10^{-3}	0.61	0.68
244	**Mitochondrial fatty acid Beta oxidation**	10	-0.07	1.8×10^{-7}	1.0×10^{-6}	-0.04	1.2×10^{-6}	5.4×10^{-6}
278	**tRNA aminoacylation**	9	-0.07	$< 10^{-7}$	$< 10^{-7}$	-0.04	$< 10^{-7}$	$< 10^{-7}$
85	Glycosphingolipid Metabolism	8	-0.07	$< 10^{-7}$	$< 10^{-7}$	-0.01	0.17	0.23
313	CDC6 association with the ORC origin complex	7	-0.08	3.3×10^{-4}	1.0×10^{-3}	0.02	0.27	0.34
221	Apoptotic cleavage of cell adhesion proteins	6	-0.08	6.0×10^{-6}	2.8×10^{-5}	0.01	0.40	0.49
257	**Cytosolic tRNA Aminoacylation**	5	-0.09	$< 10^{-7}$	$< 10^{-7}$	-0.03	$< 10^{-7}$	1.9×10^{-7}
661	**Metabolism of Porphyrins**	4	-0.10	$< 10^{-7}$	$< 10^{-7}$	-0.04	6.7×10^{-5}	2.1×10^{-4}
300	Hormone ligand binding receptors	3	-0.10	1.5×10^{-6}	7.8×10^{-6}	-0.01	0.33	0.41
558	Ethanol oxidation	2	-0.13	$< 10^{-7}$	$< 10^{-7}$	-0.01	0.46	0.55
485	Amino acid synthesis and interconversion transamination	1	-0.17	$< 10^{-7}$	$< 10^{-7}$	-0.02	0.07	0.11

Results of Quantitative Set Analysis of Gene Expression (Qusage version 2.2.2) analysis comparing gene expression values of genes assigned to the specified Reactome Pathways in *TRIB1*^{High} versus *TRIB1*^{Low} samples in CTS dataset. Ranking of Reactome pathways (version 4.0) according to MDM transcriptome. Increased (above dotted line) and reduced average RNA levels of gene set assigned to specified REACTOME Pathway. Pathways highlighted in **Green** are significantly altered in both cell types; lipid-metabolism associated pathways (only altered in macrophages are highlighted in **Red**). **357** (MDM) and **332** (monocytes) ranked pathways were returned with P values < 0.05.

Table S4. Linoleic (LiA), oleic (OA), and lauric acid (LA) in vitro polarized human MDMs recapitulate the *Olr1*^{High}/*Lpl*^{High}/*Scarb1*^{Low}/*CD36*^{WT} RNA profile *of Trib1*^{mTg} BMDM.

	OLR1		***LPL***		***SCARB1***	
Stimulus	Fold Change	p	Fold Change	p	Fold Change	p
**LiA**	**6.36**	**6.60E-09**	**2.86**	**2.65E-05**	**-1.99**	**7.68E-07**
SA	6.17	1.08E-08	3.19	4.00E-06	-1.85	8.55E-06
**OA**	**5.50**	**5.70E-12**	**2.65**	**3.87E-07**	**-1.38**	**1.66E-03**
**LA**	**5.20**	**1.73E-07**	**4.44**	**6,37E-09**	**-1.33**	**3.32E-02**
PA	4.76	1.78E-10	5.62	7.26E-17	-1.75	1.31E-07
TNF_PGE2	4.42	3.88E-04	-2.10	2.58E-02	-1.52	2.33E-02
TNF_P3C	3.86	1.21E-03	-1.38	3.30E-01	-1.24	2.34E-01
upLPS	3.42	3.40E-06	1.82	4.15E-03	-1.12	3.14E-01
TPP	2.66	9.76E-05	-2.80	5.33E-07	-1.38	3.53E-03
TPP_IFNb	2.37	3.68E-02	-3.28	4.31E-04	-1.62	8.93E-03
TNF	2.30	6.50E-03	1.86	1.12E-02	-1.68	1.61E-04
sLPS	1.86	2.10E-02	-1.36	1.60E-01	-1.27	4.43E-02
sLPS_IFNg	1.80	1.54E-01	-2.00	3.71E-02	-1.62	8.86E-03
IFNg	1.73	5.39E-02	1.33	2.10E-01	-1.51	1.17E-03
IFNg_TNF	1.49	3.30E-01	2.14	2.20E-02	-1.92	4.33E-04
sLPS_IC	1.46	3.57E-01	-1.49	2.27E-01	-1.38	8.14E-02
HDL	1.33	4.31E-01	-1.17	5.81E-01	-1.02	8.94E-01
IFNb	1.25	5.91E-01	1.45	2.58E-01	-1.07	7.25E-01
TPP_IFNb_IFNg	-1.01	9.81E-01	-3.52	1.95E-04	-1.91	4.74E-04
IL10	-1.04	9.31E-01	2.74	2.59E-03	1.09	6.24E-01
P3C_PGE2	-1.18	6.90E-01	-1.84	6.58E-02	-1.33	1.14E-01
P3C	-1.33	4.93E-01	-1.03	9.23E-01	1.00	9.91E-01
PGE2	-1.37	4.46E-01	-1.59	1.63E-01	1.08	6.75E-01
upLPS_IC	-1.44	3.76E-01	1.03	9.24E-01	1.23	2.53E-01
IL13	-1.97	9.89E-02	3.52	1.92E-04	-1.37	8.55E-02
GC	-2.28	4.59E-02	-3.33	3.62E-04	1.38	8.13E-02
IL4_upLPS	-2.37	4.74E-03	1.61	5.12E-02	1.36	2.14E-02
IL4	-3.04	1.49E-06	2.05	9.04E-05	1.03	7.69E-01
TRIB1 high MDM	1.86	4.76E-16	1.43	6.32E-11	-1.18	2.00E-08

[A]Data extracted from Xue *et al.*

Italics indicate the eleven conditions that produce reciprocal changes in OLR1 and SCARB1 RNA.

***Bold** indicates the three conditions that recapitulate the Olr1*^{High}/*Scarb1*^{Low}/*Lpl*^{High} profile of *Trib1*^{mTg} BMDMs.

Table S5. Primer sequences.

Gene	Forward primer (5'→3')	Reverse primer (5'→3')
Abca1	GGCCAGTCTGTGTAACGGAT	TGCATCGAGCTTCTTCCTCG
Abcg1	AGGTCTCAGCCTTCTAAAGTTCCTC	TCTCTCGAAGTGAATGAAATTTATCG
Abdh5	GTGTCCCCTGCACTTACAAGA	GGAGGACAAGTGGCGTCTTA
Acat1	ATTTGCTGACGCTGCTGTAGA	AAGGCTTCATTTACTTCCCACATTG
β-actin	GGGACCTGACAGACTACCTCATG	GTCACGCACGATTTCCCTCTCAGC
Cd163	CAGCGTAGTCTGCTCACGAT	AGTTGCTCCTGGCTGGTATG
Cd68	AAGGGGGCTCTTGGGAACTA	CGAAGGGATCGTCATAGCCC
Cd36	ATGGGCTGTGATCGGAACTG	GTCTTCCCAATAAGCATGTCTCC
Ces1	AGGGAGTTCTCGACGCAATG	ATGTAGGTGGGAGCTCCAGT
Cxcl16	GAGCGCAAAGAGTGTGGAACT	TGTGGACAAGGACCTGAAAAGT
Hadhb	ACCTGCGTTCATCAAACCCT	CAGAAGCGCCATCAGTCAGG
Lpl	TTGCAGAGAGAGGACTCGGA	GTTGCACCTGTATGCCTTGC
Lxrα	TCAGCATCTTCTCTGCAGACCGG	TCATTAGCATCCGTGGGAACA
Marco	GACAAGCCCTTCTTCTCGCT	CCAAGCCCTCTTTAAGCCCC
Msr1	CGCACGTGGAACAGGAAGTA	TCTGTGAGTGTTCCCAGTCC
Nceh1	AGAAGACCCTGCCACAATGA	CTGTGACTACGGGACACGATT
Olr1	AGATAGACACCCTCACCTTGAA	GTCACGCACGATTTCCCTCTCAGC
Pltp	CCATGTTGCCCGAAGTGGAG	CCCCGGAGTGTCAACTTAGC
Pparγ	GCCCTGGCAAAGCATTTGTA	TTTCCTGTCAAGATCGCCCT
Ptgs1	GTTCCGAGCCCAGTTCCAATA	AGCTGTACTCTTGTGAGCCC
Scarb1	GATGGAGAGCAAGCCTGTGA	GACTATGTGCAGGGGTGCTC
Stab1	ACGCTGACTGCCTCAATACC	AAGCATCAGTGTGGCAAGT
Lyz2Cre	Common:<br>CTTGGGCTGCCAGAATTTCTC	Mutant:<br>CCCAGAAATGCCAGATTACG<br><br>Wild type:<br>TTACAGTCGGCCAGGCTGAC
Rosa26.Trib1	Common:<br>GTGATCTGCAACTCCAGTCTTTCTAG	Mutant:<br>CCTTCTTGACGAGTTCTTCTGAGG<br><br>Wild type:<br>CGCGACACTGTAATTTCATACTGTAG
Trib1 fl/fl	Common:<br>ACCTTGATCTGCAGTCCTAGG	Floxed:<br>AAGTTCACATTTGAACTGATGGC<br><br>Wild type:<br>AGCTGGTTTCAGGGGAAGAC
